# Hypoxia Induces M2 Macrophages to Express VSIG4 and Mediate Cardiac Fibrosis After Myocardial Infarction

**DOI:** 10.7150/thno.78736

**Published:** 2023-04-02

**Authors:** Yan Wang, Yu Zhang, Jiao Li, Chaofu Li, Ranzun Zhao, Changyin Shen, Weiwei Liu, Jidong Rong, Zhenglong Wang, Junbo Ge, Bei Shi

**Affiliations:** 1 Department of Cardiology, Affiliated Hospital of Zunyi Medical University, Zunyi 563000, China; 2 Soochow University, Suzhou, China; 3 Department of Cardiology, Zhongshan Hospital, Fudan University, Shanghai Institute of Cardiovascular Diseases, Shanghai, China

**Keywords:** Myocardial infarction, Hypoxia, Macrophages, VSIG4, Cardiac fibrosis

## Abstract

M2 macrophage-mediated tissue repair plays an important role in acute myocardial infarction (AMI). Additionally, VSIG4, which is mainly expressed on tissue-resident and M2 macrophages, is crucial for the regulation of immune homeostasis; however, its effects on AMI remain unknown. In this study, we aimed to investigate the functional significance of VSIG4 in AMI using *VSIG4* knockout and adoptive bone marrow transfer chimeric models. We also determined the function of cardiac fibroblasts (CFs) through gain- or loss-of-function experiments. We showed that VSIG4 promotes scar formation and orchestrates the myocardial inflammatory response after AMI, while also promoting TGF-β1 and IL-10. Moreover, we revealed that hypoxia promotes VSIG4 expression in cultured bone marrow M2 macrophages, ultimately leading to the conversion of CFs to myofibroblasts. Our results reveal a crucial role for VSIG4 in the process of AMI in mice and provide a potential immunomodulatory therapeutic avenue for fibrosis repair after AMI.

## Introduction

Ventricular remodeling (VR) is essential for the progression from acute myocardial infarction (AMI) to heart failure 1. The proliferation, migration, and phenotypic transformation of fibroblasts play important roles in mediating tissue healing and preventing adverse VR 2. Therefore, modulating the level of myocardial fibrosis after AMI is important for delaying or reversing VR.

Macrophages are the core regulators of inflammatory responses, and as such, are involved in the regulation of cardiac inflammatory responses and tissue repair after AMI. Through in-depth exploration of macrophages, recent research has shown that the phenotype of macrophages exhibits greater plasticity than previously assumed 3. The previous classifications of M1 and M2 phenotypes represent only a continuum of two extreme activation states. Although macrophages play a role in inflammation and repair, their specific roles are related to the expression and function of their surface receptors 4-6. The activation of these surface receptors leads to the activation of signaling mediators, which transcriptionally regulate the production of pro- or anti-inflammatory mediators, including cytokines, chemokines, and adhesion molecules, thereby mediating tissue inflammation and regenerative repair processes.^4^ Therefore, treatments targeting these receptors, signaling molecules, inflammatory mediators, and cytokines can reduce myocardial injury.

V-set and immunoglobulin domain-containing 4 (VSIG4) is a complement receptor of the immunoglobulin superfamily (CRIg) that is specifically expressed in resting tissue-resident macrophages 7,8. Notably, CRIg is expressed in monocyte-derived macrophages but not in human and mouse monocytes.^7^ VSIG4 can suppress T cell proliferation and promote Foxp3^+^ regulatory T cell (Tregs) differentiation by binding an unidentified receptor on T cells 9. The VSIG4-Fc fusion protein protects against the development of experimental autoimmune arthritis, uveoretinitis, and hepatitis 10. Importantly, VSIG4 is significantly reduced after myocardial ischemia-reperfusion injury in patients undergoing coronary artery bypass grafting 11. In contrast, in patients with chronic right heart failure, serum VSIG4 expression is increased and may be a molecular marker of right heart failure 12. Nevertheless, whether VSIG4 is expressed in cardiac-infiltrating macrophages after AMI and whether it is involved in macrophage-mediated regulation of cardiac fibrosis, as well as its related mechanisms, remain unclear.

In the present study, we aimed to first observe the changes in VSIG4 expression at different time points following myocardial infarction. Subsequently, the area of myocardial infarction and cardiac function was observed in VSIG4-knockout mice to clarify whether VSIG4 plays a role in tissue fibrosis repair following myocardial infarction. Immediately afterwards, a bone marrow transmigration assay was conducted to determine whether cardiac-resident macrophages or bone marrow-derived M2 macrophages expressed VSIG4 while regulating fibrotic repair following myocardial infarction. Then, the effect of VSIG4-expressing bone marrow M2 macrophages on the proliferation, migration, and phenotypic conversion of cardiac fibroblasts (CFs) to myofibroblasts was observed in in-vitro experiments to explore the mechanisms associated with VSIG4 expression by M2-type macrophages. Our findings enhance our understanding of the functions and mechanisms of action of the immune co-suppressor molecule VSIG4 in bone marrow-derived M2 macrophage-mediated fibrotic repair following myocardial infarction. This study provides an experimental theoretical basis for understanding the role of macrophages and their receptor expression in myocardial infarction.

## Methods

The data, analytical methods, and study materials are available from the corresponding author upon reasonable request.

Detailed Materials and Methods are provided in the online Supplemental Material.

### Mice

All animal procedures conformed to the Guide for the Care and Use of Laboratory Animals, published by the US National Institutes of Health (NIH publication no. 85-23, revised 1996). Animal care, surgery, and handling procedures were approved by the Institutional Animal Care and Use Committee of the University of Zunyi Medical University.

VSIG4^-/-^ (gene symbol: CRIg; VSIG4KO) mice with a C57BL/6 background were generated as previously described 13. Wild-type (WT) C57BL6/J mice (20-25 g, male, 6-16 weeks old; Laboratory Animal of Zunyi Medical University, Zunyi, China) were used as controls.

### Additional Methods

The expanded Methods section in the online Supplemental Material includes information on the treatment and group, the description of flow cytometric analyses, quantification of absolute cell numbers, quantitative real-time polymerase chain reaction (PCR), echocardiographic analyses of cardiac function, infarct size assessment, western blotting, bone marrow transplantation, cell culture, and enzyme-linked immunosorbent assay. All antibodies and kits used in this study are listed in Supplemental Tables S1 and S2.

### Statistical Analysis

Statistical analysis was mainly performed using SPSS 21.0 statistical software package (IBM, Armonk, NY, USA) and GraphPad Prism 7.0 (GraphPad Software Inc., San Diego, CA, USA). The data were normally distributed and are expressed as the mean ± SD. Comparisons between two groups were made using the Mann-Whitney U test, whereas one-way analysis of variance was used to compare multiple groups. Statistical significance was set as *P*<0.05.

## Results

### VSIG4 Levels are Increased in Macrophages in the Late Stage (D7) After AMI

An AMI model was established by ligating the left anterior descending branch of the coronary artery in mice. Electrocardiography, TTC staining, and Masson staining were performed to assess the success of the model establishment (Figure S1A). Bone marrow-derived macrophages were sorted by flow cytometry on days 3 and 7 after myocardial infarction. The infiltration of M1-type macrophages was predominant 3 days after infarction, whereas that of M2-type macrophages was predominant 7 days after infarction (Figure S1B).

**Figure 1:**
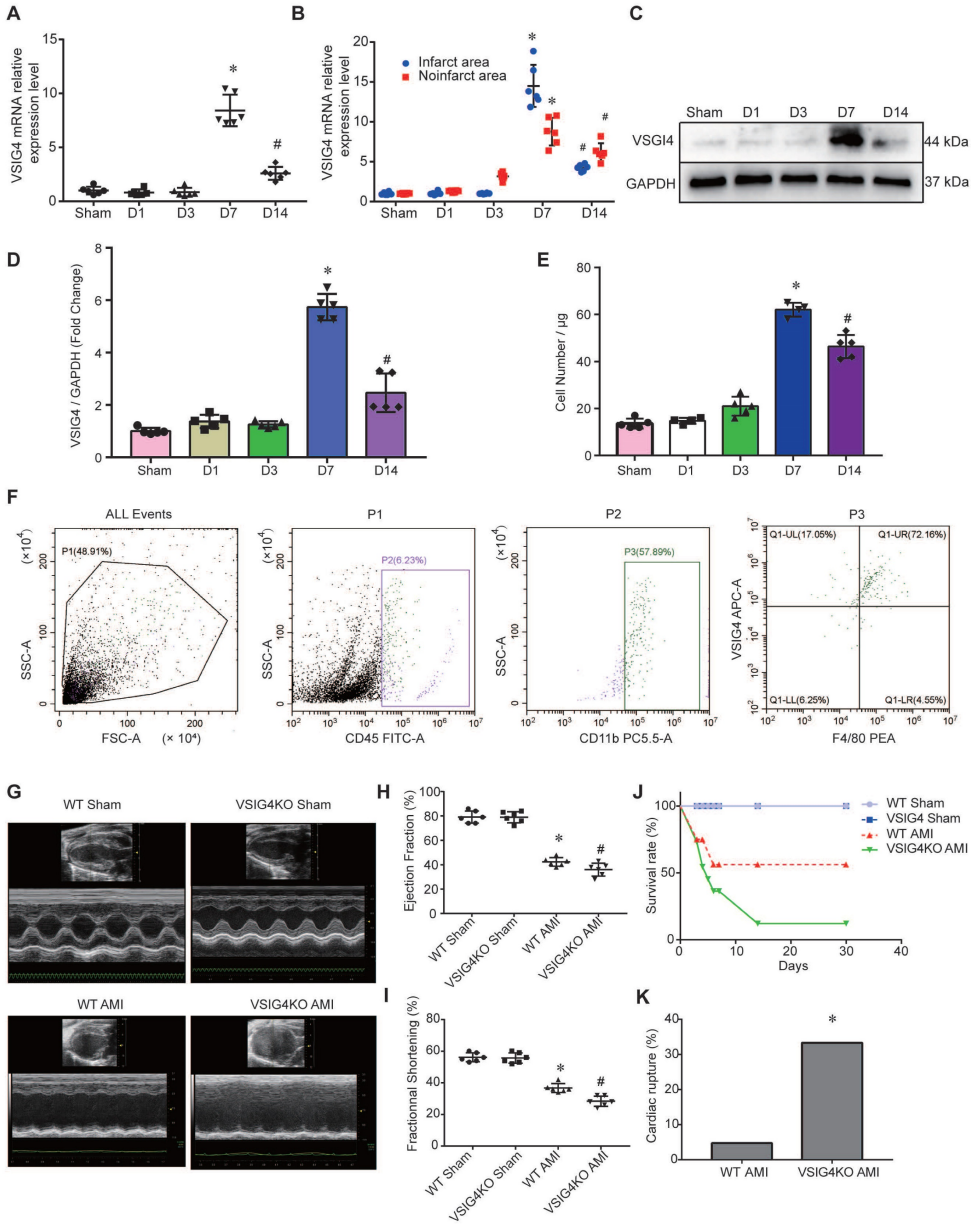
** Changes in VSIG4 expression in mouse cardiac tissue after acute myocardial infarction (AMI) and the effects of VSIG4 deficiency on cardiac function, survival, and ventricular rupture. A,** VSIG4 mRNA expression in macrophages isolated from mouse hearts at different time points after AMI (n=6, ^*^P<0.05 vs. sham group, ^#^*P<*0.05 vs. D7 group). **B,** VSIG4 mRNA expression levels in the infarcted and non-infarcted regions at different time points after AMI (n=6, ^*^P<0.05 vs. the sham group, ^#^*P<*0.05 vs. the D7 group). **C-D,** VSIG4 protein expression at different time points in the heart (n=6, ^*^P<0.05 vs. the sham group, ^#^*P*<0.05 vs. the D7 group). **E,** Temporal dynamics of VSIG4^+^ macrophage count in myocardial tissue per μg of infarct area (n=5, ^*^P<0.05 vs. the sham group, ^#^*P*<0.05 vs. the D7 group). **F,** Representative flow cytometry scatter plot illustrating the gating strategy: CD45^+^ leukocyte population, CD11b^+^ myeloid cells, and VSIG4^+^/F4/80^+^ macrophages. **G-I,** Echocardiographic analysis of fractional shortening (FS) and ejection fraction (EF) on day 28 after AMI or sham operation (n=6). **J,** Kaplan-Meier survival analysis at different time points after AMI time (n=6, ^*^P<0.05 vs. the sham group). **K,** A histogram showing the ventricular rupture rate in wild-type (WT) vs. *VSIG4*-knockout (VSIG4KO) mice after AMI (n=6, ^*^P<0.05 vs. the WT AMI group).

To assess the changes in VSIG4 expression in macrophages after myocardial infarction, we screened macrophages by flow cytometry on days (D) 1, 3, 7, and 14 after myocardial infarction. We detected VSIG4 mRNA expression after RNA extraction and found that VSIG4 was significantly more highly expressed on D7 (*P*<0.05) and significantly less expressed on D14 (*P*<0.05) after myocardial infarction (Figure 1A). Additionally, VSIG4 mRNA expression was significantly higher on D7 after AMI than that in the sham group and was significantly decreased on D1, D3, and even D14 after AMI (*P*<0.05). In addition, VSIG4 protein and mRNA expression in the infarcted area was significantly higher than that in the non-infarcted area of WT mice (Figure 1B). Western blotting results also showed that VSIG4 protein expression was significantly increased on D7 after AMI (*P*<0.05; Figure 1C and 1D).

To detect the number of VSIG4^+^ macrophages infiltrating the myocardium at different time points of AMI, we performed double-immunofluorescence staining of VSIG4 and F4/80. The results confirmed that VSIG4^+^/F4/80^+^ macrophages were significantly more abundant in the D7 group than in the sham, D1, and D3 groups. Notably, the number of VSIG4^+^/F4/80^+^ macrophages was lower in the D14 group than in the D7 group (*P*<0.05; Figure 1E and 1F).

To further clarify how M1/M2 cardiac macrophage change after MI and whether VSIG4 is explicitly expressed in M2 macrophages or also expressed and has functions in M1 macrophages, we performed Western blot assays in the sham-operated group, on days 3-7 after MI, and showed that VSIG4 was significantly highly expressed in myocardial tissue on day 7 of the infarction (Figure S2A). Subsequently, immunofluorescence and flow-through cytometry were used to further clarify the expression of VSIG4 in M1 and M2 macrophages after myocardial infarction. The results showed that VSIG4^+^CD206^+^ macrophages were progressively more highly expressed on days 3-7 post-infarction and that more than 60% of M2 macrophages expressed VSIG4 on day 7 of infarction (Figure S2B and S2D), whereas VSIG4 was barely expressed in M1 macrophages (Figure S2C and S2E). These results indicate that macrophages infiltrating the cardiac tissue highly express VSIG4 7 days after AMI. To verify what effect the absence of VSIG4 has on the infiltration of M1/M2 macrophages.We examined the infiltration of M1/M2 macrophages in VSIG4KO mice after myocardial infarction. The results showed that the M1/M2 macrophages in VSIG4KO mice at day 3 and day 7 of infarction were consistent with those in WT mice (Figure S3). This indicates that VSIG4 gene deletion does not lead to changes in infiltration of M1 and M2 macrophages after myocardial infarction.

### VSIG4 Knockout Inhibits Wound Healing and Scar Formation After AMI

To elucidate the effect of VSIG4 on AMI, we generated VSIG4 knockout mice (VSIG4KO) and compared the severity of AMI in these mice to that in WT C57BL/6 mice. Prior to the surgery, we excluded the possibility of any cardiac abnormalities in VSIG4KO mice by performing echocardiography, and VSIG4KO mice had echocardiographic parameters measured similar to those of the WT mice. Echocardiographic analysis of cardiac function in mice 28 days after AMI showed that ejection fraction and left ventricular fractional shortening were significantly lower in VSIG4KO mice after AMI compared to WT mice (Figure 1G-1I). We also showed that the survival rate was lower in VSIG4KO mice than in WT mice on D28 after AMI, whereas all sham-operated animals in both groups survived until the end of the study (Figure 1J). The incidence of cardiac rupture-induced death was significantly higher in VSIG4KO mice than in WT mice after AMI (Figure 1K).

**Figure 2:**
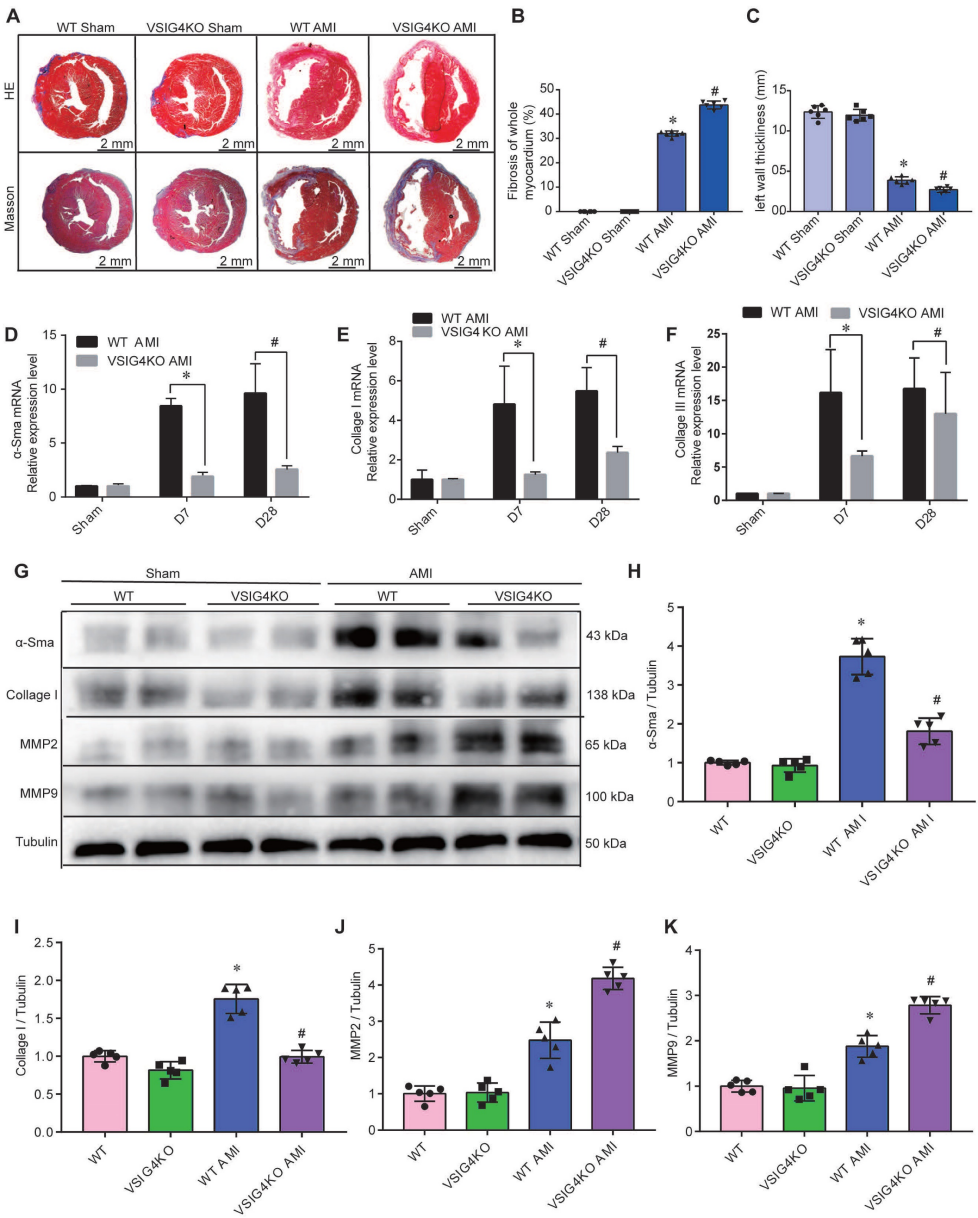
***VSIG4* knockout inhibits wound healing and scar formation after acute myocardial infarction (AMI). A,** Representative Masson trichrome staining and hematoxylin and eosin (HE staining) of cardiac tissue obtained from wild-type (WT) or *VSIG4*-knockout (VSIG4KO) mice 28 days after AMI or sham operation. **B-C,** Quantitative analysis of infarct size and wall thickness 28 days after AMI in WT or VSIG4KO mice after Sham or AMI operation (n=6, ^*^P<0.05 vs. the WT group,^ #^*P*<0.05 vs. the WT AMI group).** D-F**, Gene expression levels of collagen I, collagen III, and α-SMA (smooth muscle actin) at different time points after AMI or sham operation in WT and VSIG4KO mice (n=4-5, ^*^P<0.05 vs. the WT AMI-D7 group,^ #^*P*<0.05 vs. the WT AMI-D28 group). **G-K,** Protein expression levels of collagen I, α-SMA, matrix metallopeptidase (MMP) 2, and MMP9 in the scar tissue isolated from WT and VSIG4KO mice 7 days after AMI (n=5, ^*^P<0.05 vs. the WT group,^ #^*P*<0.05 vs. the WT AMI group).

Hematoxylin and eosin and Masson's trichrome staining showed no difference in the cardiac gross morphology of the sham-operated hearts between WT and VSIG4KO mice (Figure 2A-2C). VSIG4KO mice showed a significant increase in the myocardial infarct area and a significant decrease in ventricular wall thickness in the infarcted area on D28 after AMI, compared to WT mice. We subsequently analyzed myocardial wound healing and myofibroblast differentiation parameters after AMI to understand the mechanisms underlying the cardiac rupture. We found that α-smooth muscle actin (α-SMA), collagen I, and collagen III expressions in the infarcted heart were remarkably higher in WT mice than in VSIG4KO mice on D7 and D28 (Figure 2D and 2E). The protein levels of α-SMA and collagen I were also significantly higher in WT mice than in VSIG4KO mice on D7 after AMI (Figure 2G-2I). Additionally, we assessed matrix metallopeptidase (MMP) 2 and MMP9 expression levels in the heart on D7 after AMI. We observed significantly increased MMP2 and MPP9 expression levels in the infarct areas of VSIG4KO mice on D7 after AMI (Figure 2G, 2J, and 2K), indicating that AMI leads to the upregulation of both MMP2 and MMP9 expression. These results indicate that VSIG4 downregulation inhibits wound healing and scar formation after AMI.

### VSIG4 is Expressed in Bone Marrow-Derived M2 Macrophages and Mediates Wound Healing and Scar Formation After AMI

**Figure 3:**
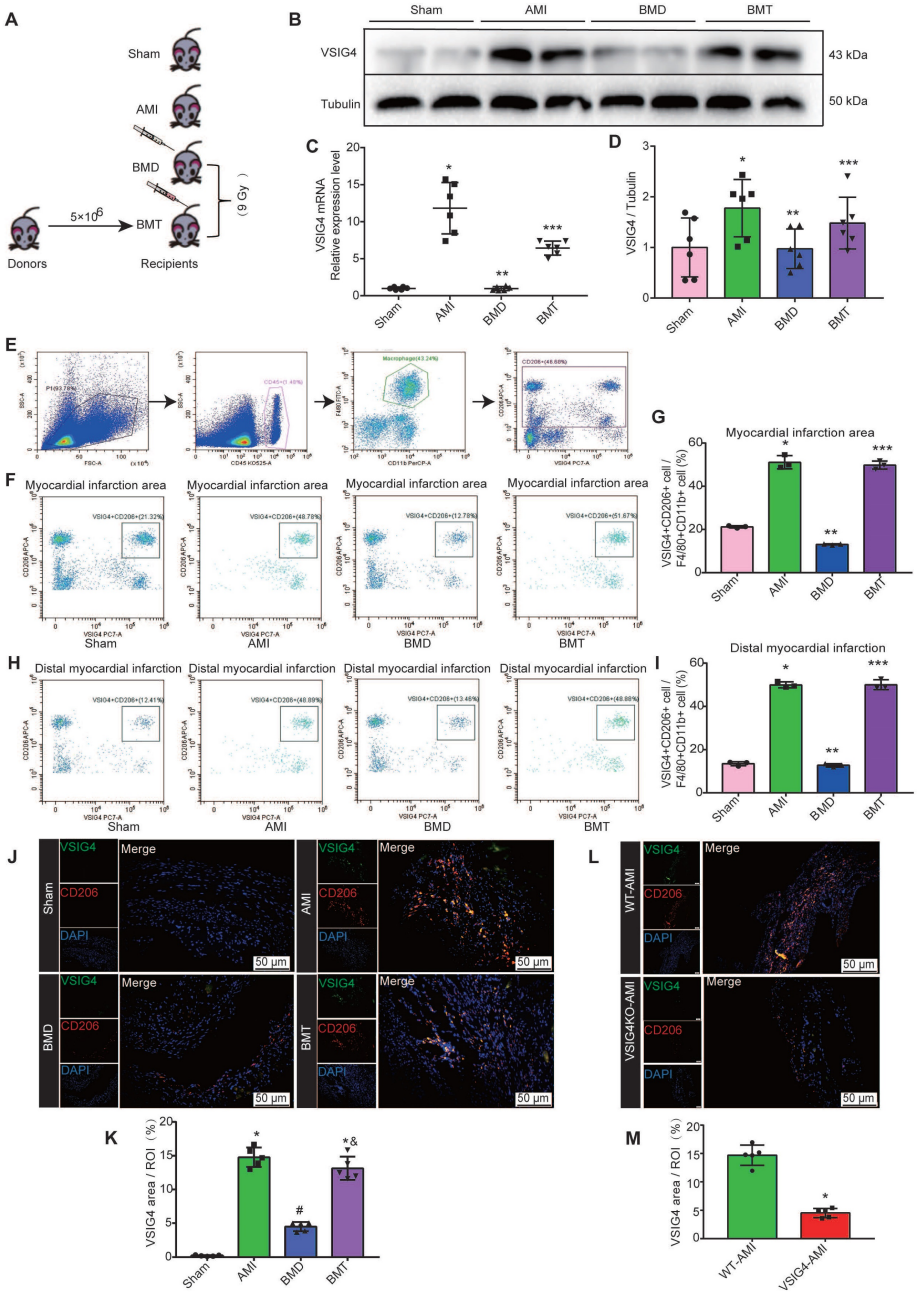
** Bone marrow-derived macrophages are major contributors to VSIG4-positive M2 macrophages in cardiac tissue after acute myocardial infarction (AMI). A,** Schematic diagram of experimental grouping for preparation of AMI model after adoptive transfer of wild-type (WT) mouse bone marrow.** B-C,** Western blotting for VSIG4 protein expression in the scar tissue isolated from BMD (bone marrow depletion) and BMT (bone marrow transplant) mice 7 days after AMI. **D,** VSIG4 mRNA expression levels in different groups (n=6, ^*^P<0.05 vs. the sham group,^ **^P<0.05 vs. the AMI group,^ ***^P<0.05 vs. the BMD group). **E,** Representative flow cytometric analysis of VSIG4 expression on gated CD11b^+^ myeloid cells and F4/80^+^ macrophages in the heart on day 7 after AMI. **F,** Representative flow cytometric dot plots of VSIG4 expression on gated CD11b^+^F4/80^+^CD206^+^ macrophages in the infarct zone of different groups after AMI. **G,** The ratio of VSIG4^+^CD206^+^ macrophages to CD11b^+^F480^+^ macrophages in the heart tissue (n=3, ^*^P<0.05 vs. the sham group,^ **^P<0.05 vs. the AMI group,^ ***^P<0.05 vs. the BMD group). **H,** Representative flow cytometric dot plots of VSIG4 expression on gated CD11b^+^ F4/80^+^CD206^+^ macrophages in the distal infarction in different groups after AMI. **I,** The ratio of VSIG4^+^CD206^+^ macrophages to CD11b^+^F480^+^ macrophages in the heart tissue (n=3, ^*^P<0.05 vs. the sham group,^ **^P<0.05 vs. the AMI group,^ ***^P<0.05 vs. the BMD group). **J,** Representative dual-immunofluorescence staining for VSIG4 and CD206 in the heart on day 7 after AMI in different groups (Scale bar, 50 μm). **K,** A histogram showing VSIG4 fluorescence intensity (n=5, ^*^P<0.05 vs. the sham group,^ #^*P*<0.05 vs. the AMI group,^ &^*P*<0.05 vs. the BMD group). **L,** Representative dual-immunofluorescence staining for VSIG4 and CD206 in the heart of mice 7 days after AMI in different groups (Scale bar, 50 μm). **M,** Histogram showing VSIG4 fluorescence intensity (n=5, ^*^P<0.05 vs. the WT AMI group).

VSIG4 was previously shown to be mainly expressed in tissue-resident cells 8; however, both cardiac-resident and bone marrow-infiltrating macrophages play an important role in cardiac repair after AMI. Therefore, we aimed to investigate whether cardiac-resident or bone marrow-derived express VSIG4 to regulate fibrotic repair after AMI by performing bone marrow macrophage depletion (BMD) and bone marrow cell transplantation (BMT) in WT mice (Figure 3A). Real-time quantitative PCR (RT-qPCR) and western blotting showed that BMD significantly reduced VSIG4 expression in cardiac tissues after AMI, and BMT rescued the decrease in VSIG4 at the mRNA and protein levels (Figure 3B-3D). Since VSIG4 was mainly expressed in myocardial infarct area tissue on D7 after AMI, and this time point was dominated by CD206-positive M2 macrophage infiltration, we used flow cytometry and immunofluorescence double staining to further clarify whether VSIG4 was expressed in CD206-positive bone marrow-derived macrophages. The results showed that BMD remarkably decreased the number of VSIG4^+^CD206^+^ macrophages that infiltrated the myocardial infarction area after AMI, whereas BMT restored VSIG4 expression and the infiltration of VSIG4^+^CD206^+^ macrophages after AMI (Figure 3E-3J). In addition, VSIG4+CD206+ macrophage infiltration was significantly reduced in the cardiac tissue of VSIG4KO mice compared to that in WT mice after AMI (Figure 3L and 3M). Notably, BMD resulted in enlarged myocardial infarct size and thinning of the left ventricular wall in mice on D28 after AMI. BMT reversed the decrease in α-SMA, collagen I, and collagen III expression levels and the increase in MMP2 and MMP9 expression levels observed in BMD mice on D7 after AMI (Figure S4A-S4C). These results showed, at least in part, the involvement of VSIG4-expressing bone marrow-derived M2 macrophages in the regulation of fibrosis after AMI.

**Figure 4:**
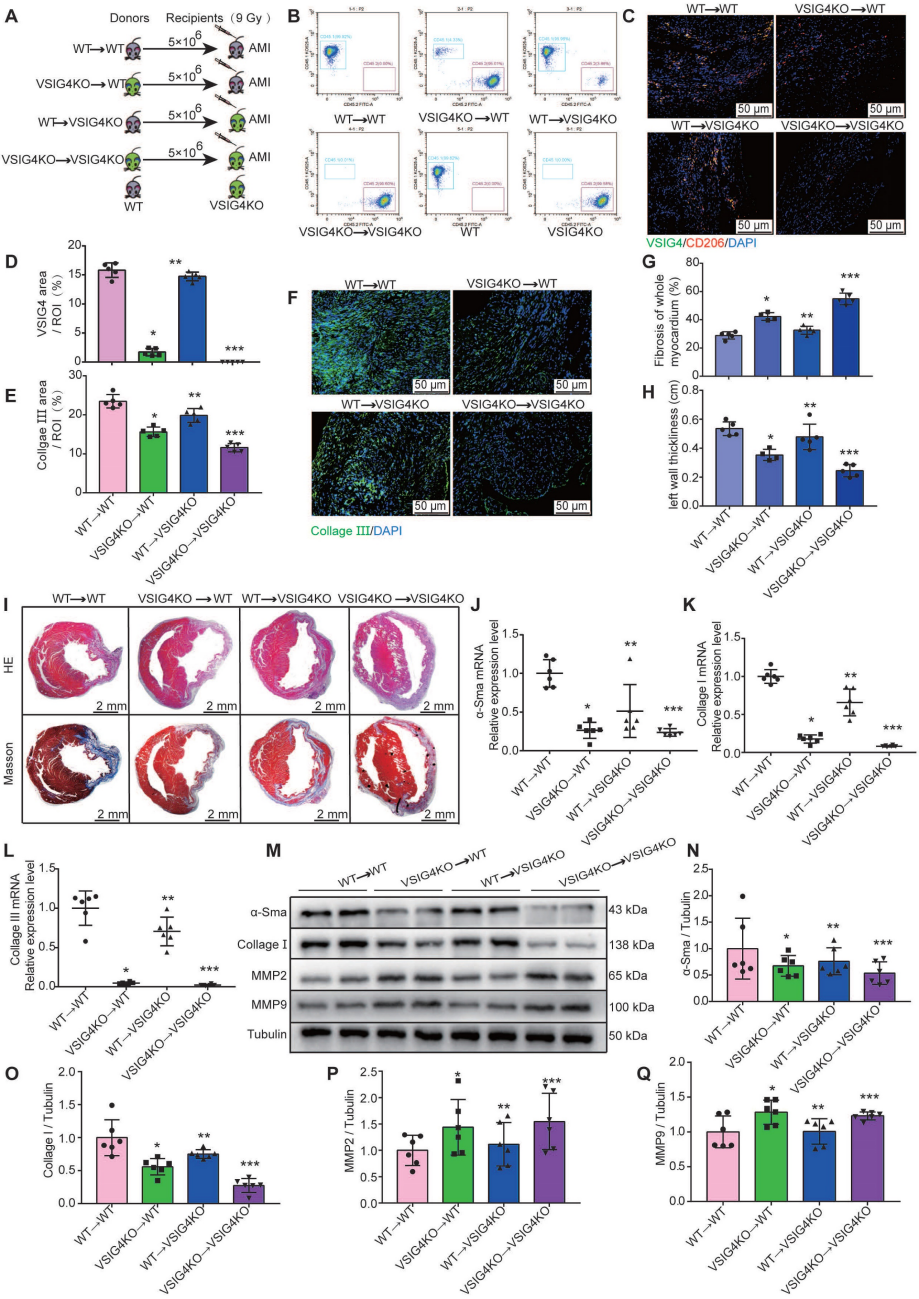
** VSIG4-expressing bone marrow-derived M2 macrophages regulate fibrosis after acute myocardial infarction (AMI). A,** Experimental setup. Bone marrow transplantations (BMTs) were performed between wild-type (WT) and VSIG4-knockout (VSIG4KO) mice. Mice were subjected to AMI 6 weeks after BMT. The following groups of mice were studied: WT→WT, VSIG4KO→WT, WT→VSIG4KO, and VSIG4KO→VSIG4KO. **B,** Flow cytometric analysis of peripheral blood cells to confirm successful BMTs between CD45.1 (WT) and CD45.2 (VSIG4KO) mice (n=3). Flow cytometric analysis showing the genetic background of CD45 alleles in WT and VSIG4KO mice (n=3).** C,** Representative dual-immunofluorescence staining for VSIG4 and CD206 in the heart on day 7 after AMI in different groups (Scale bar, 50 μm). **D,** Histogram showing VSIG4 fluorescence intensity. **E**, Histogram of relative collagen III expression levels in each group. **F**, Representative immunofluorescent images for collagen III (green) in each group (Scale bar, 50 μm).** G-H,** Quantitative analysis of infarct size and wall thickness on day 28 after AMI in each group. **I**, Representative Masson trichrome staining and hematoxylin and eosin (HE) staining of cardiac tissue obtained from recipient WT or VSIG4KO mice on day 28 after AMI (Scale bar, 2 mm). **J-L,** Gene expression levels of collagen I, collagen III, and α-SMA (smooth muscle actin) on day 7 after AMI in the scar tissue isolated from the WT→WT, VSIG4KO→WT, WT→VSIG4KO, and VSIG4KO→VSIG4KO groups.** M-Q**, Protein expression levels of collagen I, α-SMA, matrix metallopeptidase (MMP) 2, and MMP9 on day 7 after AMI in the scar tissue isolated from the WT→WT, VSIG4KO→WT, WT→VSIG4KO, and VSIG4KO→VSIG4KO groups (n=5-6, ^*^P<0.05 vs. the WT→WT group, ^**^P<0.05 vs. the VSIG4KO→WT group, ^***^P<0.05 vs. the WT→VSIG4KO group).

To further investigate the importance of VSIG4 expression in M2 macrophages in AMI, we performed BMTs between VSIG4KO and WT mice (Figure 4A). VSIG4KO mice had a C57B/6 background expressing CD45.2; thus, we used a substrain of C57B/6 mice expressing the CD45.1 allele (C57B/6-CD45.1), similar to the WT mice, to facilitate monitoring. Flow cytometric analyses confirmed that WT C57B/6-CD45.1 (CD45.1-WT) mice expressed the CD45.1 allele, whereas VSIG4KO mice expressed CD45.2 (Figure 4B). Six weeks after BMT, we demonstrated that the bone marrow of the recipient mice was successfully reconstituted with donor bone marrow cells by analyzing peripheral blood cells using flow cytometry (Figure 4B). Recipient mice that underwent successful BMT were subjected to AMI, and we further showed that the bone marrow-derived donor cells infiltrated into the heart and expressed VSIG4 and CD206 on D7 after AMI using immunofluorescence staining (Figure 4C and 4D).

We assessed the extent of myocardial fibrosis on D7 and analyzed the area of myocardial infarction and ventricular wall thickness on D28 in the recipient mice. The transplantation of VSIG4KO bone marrow-derived cells to WT mice (VSIG4KO→WT) led to a significant decrease in collagen III expression levels and ventricular wall thickness and a significant increase in the infarct size compared to WT mice transplanted with WT bone marrow (WT→WT). In contrast, transplantation of WT bone marrow-derived cells into VSIG4KO mice (WT →VSIG4KO) resulted in a considerable increase in collagen III expression levels and ventricular wall thickness, and a decrease in the infarct size, compared to VSIG4KO mice transplanted with VSIG4KO bone marrow-derived cells (VSIG4KO→VSIG4KO) (Figure 4E-4I). Consistent with these findings, we observed impaired cardiac fibrosis healing, as indicated by lower α-SMA, collagen I, and collagen III expression levels and higher MMP2 and MMP9 expression levels in the VSIG4KO→WT group on D7 after AMI, compared to those measured in the WT→WT mice. However, cardiac function (characterized by the above parameters) was significantly improved in VSIG4KO mice transplanted with WT bone marrow (Figure 4J-4Q). These results indicate that VSIG4 expressed in bone marrow-derived M2 macrophages critically contributes to AMI.

### Hypoxia Induces M2 Macrophages to Express VSIG4 and Mediate the Proliferation, Migration, and Phenotypic Transformation of Cardiac Fibroblasts

We observed that *in vitro*-cultured bone marrow M2 macrophages expressed higher VSIG4 mRNA levels than did M0 and M1 macrophages; however, there was no significant difference in VSIG4 protein levels among the three groups (Figure S5A-S5C). Permanent coronary artery ligature the blood flow is completely abolished, giveing rise to a relatively hypoxic environment at the site of myocardial infarction 14. Additionally, VSIG4 is a surface marker of M2 macrophages in hypoxic tumor tissues 15. Therefore, we investigated whether hypoxia mediates the fine-tuned VSIG4 expression in bone marrow-derived M2 macrophages by assessing the viability of M2 macrophages under hypoxic conditions at various time points using cell counting kit 8 (CKK8) assays.

We observed an increase in M2 macrophage viability with increasing hypoxia time, with a significant increase after 24 h of hypoxia; however, the difference was not statistically significant. Notably, when the hypoxia time was increased to 48 h, cell viability decreased significantly. Therefore, 24 h of hypoxia was selected for the subsequent experiments (Figure S5C). RT-qPCR and western blotting revealed that 24 h of hypoxia effectively promoted the upregulation of VSIG4 expression compared to the normoxic group (Nor) (Figure S5D and S5E). Subsequently, we detected the cytokine released from M2 macrophages and cell morphology and observed that the 24 h-hypoxic condition did not affect the M2 macrophage phenotype (Figure S5F and S5K). However, EdU staining results showed that 24 h-hypoxia induced M2 macrophage proliferation (Figure S5L).

We cultured and identified CFs by staining for discoidin domain receptor 2 (DDR2) and vimentin (Figure S5M), and overexpressed or inhibited VSIG4 in M2 macrophages using adenovirus transfection (Figure S5N). We subsequently established a co-culture system of M2 macrophages and CFs under different culture conditions (Figure S6A) to observe whether VSIG4-expressing M2 macrophages play a regulatory role. The results showed that M2 macrophages can promote the proliferation, migration, and phenotypic transformation of CFs to myofibroblasts, and these effects were significantly enhanced by M2 macrophages overexpressing VSIG4 (Figure S6B-6K). Importantly, the expression of TGF-β and IL-10, the cytokines that mediate the proliferation, migration, and phenotypic transformation of CFs, was significantly increased in the co-culture system of M2 macrophages overexpressing VSIG4 (Figure S6L and S6M).

**Figure 5:**
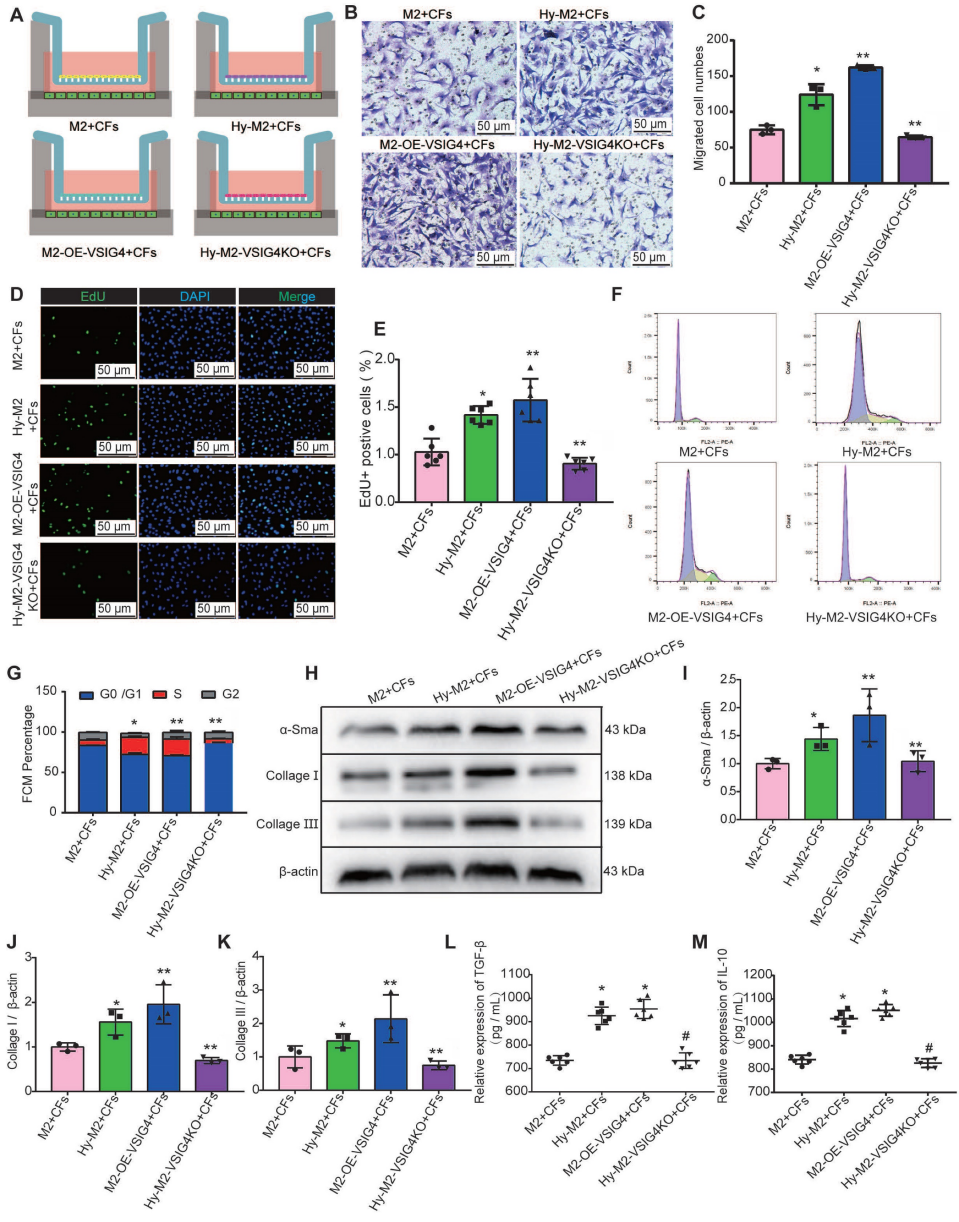
** Effect of hypoxia-mediated VSIG4 expression in M2 macrophages on proliferation, migration, and phenotypic transformation of cardiac fibroblasts. A,** Experimental setup. Hypoxic M2 macrophages (Hy-M2) or macrophages overexpressing VSIG4 (M2-OE-VSIG4) were co-cultured with cardiac fibroblasts (CFs). VSIG4-deficient M2 macrophages were cultured under hypoxic conditions for 24 h and then co-cultured with CFs. **B,** Representative images of crystal violet staining for cell migration (Scale bar, 50 μm). **C,** Quantitative analysis of migrating cells (n=3, ^*^P<0.05 vs. the M2+CFs group, ^**^P<0.05 vs. the Hy-M2+CFs group). **D,** Representative EdU fluorescent images (Scale bar, 50 μm). **E,** Quantitative analysis of the proportion of EdU-positive cells (n=6, ^*^P<0.05 vs. the M2+CFs group, ^**^P<0.05 vs. the Hy-M2+CFs group).** F,** Representative images of the cell cycle analysis. **G,** Quantitative analysis of cells in the G0/G1, S, and G2 phases in each group (n=3, ^*^P<0.05 vs. the M2+CFs group, ^**^P<0.05 vs. the Hy-M2+CFs group). **H-K,** Representative western blot results of three independent experiments and quantification of α-SMA (smooth muscle actin), collagen I, and collagen III expressions in the M2+CFs, Hy-M2+CFs, M2-OE-VSIG4+CFs, and Hy-M2-VSIG4KO+CFs groups (n=3, ^*^P<0.05 vs. the M2+CFs group, ^**^P<0.05 vs. the Hy-M2+CFs group). **L,** Quantification of TGF-β expression in the cell co-culture medium. **M,** Quantification of IL-10 expression in the cell co-culture medium (n=6, ^*^P<0.05 vs. the M2+CFs group, ^#^*P*<0.05 vs. the Hy-M2+CFs group).

We further investigated whether hypoxia regulates CFs by mediating VSIG4 expression in M2 macrophages. Hypoxic M2 macrophages (Hy-M2) and hypoxic VSIG4-deficient M2 macrophages (Hy-M2-VSIG4KO) were co-cultured with CFs (Figure 5A). The results showed that Hy-M2 macrophages had a stronger ability to promote the proliferation, migration, and collagen expression in CFs than M2 macrophages. However, the ability of Hy-M2 to mediate these effects was significantly weaker after VSIG4KO (Figure 5B-5K). We also used immunofluorescence to detect the expression of α-SMA, collagen I, and collagen III in CFs in the co-culture system. The results showed that Hy-M2 and M2 macrophages overexpressing VSIG4 promoted the expression of α-SMA, collagen I, and collagen III in CFs; however, this effect was significantly diminished in Hy-M2 macrophages compared to those overexpressing VSIG4 (Figure S7). Additionally, the expression levels of TGF-β and IL-10 were also significantly increased in the co-culture system of Hy-M2 macrophages. However, the ability of Hy-M2 to mediate the release of TGF-β and IL-10 from M2 macrophages was significantly decreased after VSIG4 knockout (Figure 5K and 5L). These results suggest that hypoxia may mediate VSIG4 expression by promoting the proliferation of bone marrow-derived M2 macrophages and secretion of TGF-β and IL-10, thereby promoting the proliferation, migration, and conversion of cultured CFs to myofibroblasts *in vitro*.

### Hypoxia Induces M2 Macrophages to Express Hif1α to Mediate VSIG4 Expression

Cells sense hypoxic signaling stimuli during oxygen deficiency, which activates the hypoxic signaling pathway, initiates downstream gene expression, and regulates cellular adaptation in the hypoxic microenvironment. Previous studies have shown that tissue regeneration after salamander and zebrafish injury is dependent on reciprocal regulation between macrophages and fibroblasts infiltrating the injury site 16,17. In addition, the hypoxic environment has been reported to promote tumor microenvironment angiogenesis by upregulating HIF-1α expression in macrophages to increase the production of vascular growth factors 18. However, the mechanism by which hypoxia induces VSIG4 expression in M2 macrophages is unclear. Therefore, we further explored the mechanism of hypoxia-mediated VSIG4 expression in bone marrow-derived M2 macrophages.

**Figure 6:**
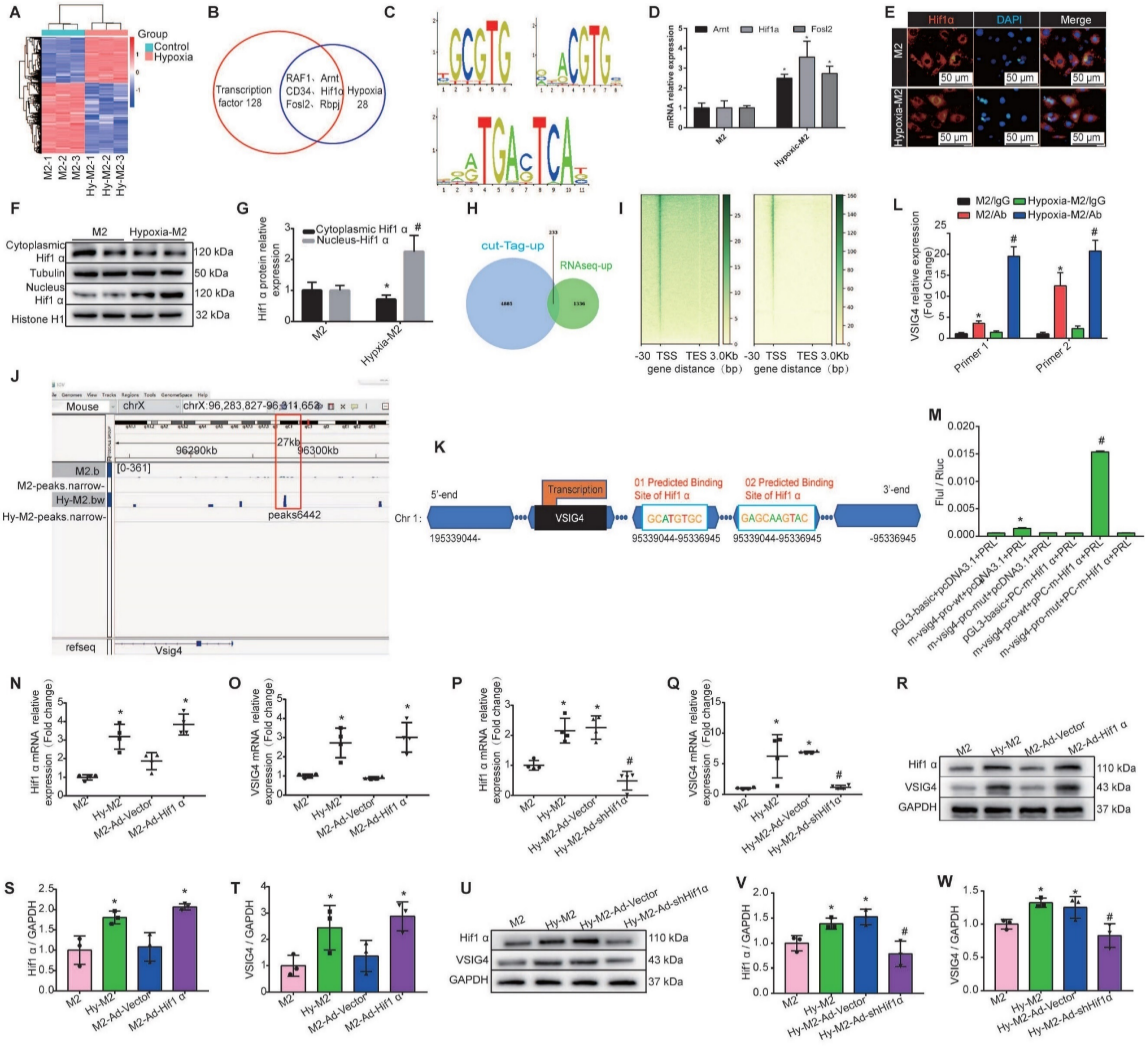
** Hypoxia induces M2 macrophages to express Hif1α and mediates VSIG4 expression. A,** A heat map showing the significantly different mRNA expression levels (*P<*0.05 and |fold change (FC)|> 2.0). **B,** Venn diagram showing the transcription factors related to transcriptional regulation and hypoxia among genes upregulated by hypoxic-M2 (Hy-M2).** C**. The motifs of Arnt, Hif1α, and Fosl2. **D,** RT-qPCR analysis showing the relative expression levels of Arnt, Hif1α, and Fosl2 in M2 and Hy-M2 (n=3, ^*^P<0.05 vs. the M2 group).** E,** Immunofluorescent images of the nuclear and cytoplasmic distribution of Hif1α in M2 and Hy-M2. Red color indicates Hif1α and blue color (DAPI) indicates chromatin. **F-G,** Western blot showing the nuclear and cytoplasmic distribution of Hif1α in M2 and Hy-M2. Anti-tubulin (Tubulin) and histone H1 (Histone H1) were used as cytoplasmic and nuclear protein internal controls, respectively (n=3, ^*^P<0.05 vs. the M2 group, ^#^*P*<0.05 vs. the cytoplasmic control protein). **H,** Venn diagram showing upregulated genes in CUT&tag and RNA sequencing analyses of normoxic and hypoxic M2 macrophages.** I,** A heatmap showing the distribution of VSIG4 binding sites detected by CUT&tag assay in M2. TSS, transcription start site; TES, transcription end site.** J,** Illustration showing Hif1α binding to the VSIG4 promoter in M2 macrophages**. K,** Schematic diagram of the putative Hif1α binding sites in the promoter regions of VSIG4. **L,** CUT&tag-qPCR analysis showing the enrichment of VSIG4 at the *Hif1α* gene in normal and hypoxic macrophages (n=3; ^*^p<0.05 vs. Hif1α antibody). **M,** Luciferase reporter assay analysis showing the VISG5 promoter activity. HEK293 cells were co-transfected with Hif1α-FLAG, wild-type (WT), or mutant VSIG4 promoter-pGL3 prior to the assay (n=3; ^*^P<0.05 vs. pGL3-basic-NC+pcDNA3.1-NC, ^#^*P<*0.05 vs. m-VSIG4-pro-WT+pcDNA3.1-NC).** N-O,** RT-qPCR showing relative mRNA levels of Hif1α and VSIG4 normalized to the GAPDH level in different groups (n=3, ^*^P<0.05 vs. the M2 group)**. P-Q,** RT-qPCR showing the relative mRNA levels of Hif1α and VSIG4 normalized to the GAPDH level in different groups (n=3, ^*^P<0.05 vs. the M2 group, ^#^*P*<0.05 vs. the Hy-M2 group). **R-T,** Representative western blot results of three independent experiments and quantification of Hif1α and VSIG4 expression in M2 macrophages under different culture conditions (n=3, ^*^P<0.05 vs. the M2 group). **U-W,** Representative western blot results of three independent experiments and quantification of Hif1α and VSIG4 expression in M2 macrophages under different culture conditions (n=3, ^*^P<0.05 vs. the M2 group, ^#^*P*<0.05 vs. the Hy-M2 group).

We first performed mRNA sequencing of *in vitro*-cultured normoxic and hypoxic bone marrow-derived M2 macrophages. We identified 1569 upregulated and 2410 downregulated genes in hypoxic M2 compared to the normoxic controls (Figure 6A). Among the 1569 upregulated genes in hypoxic M2, the molecular function of 128 transcription factors (TFs) was screened, revealing that 28 TFs were associated with hypoxia, including RAF1, hypoxia-inducible factor β (Arnt, aryl hydrocarbon receptor nuclear translocator, CD34, Hif1α (hypoxia-inducible factor α), Fosl2 (FOS like 1, AP-1 TF subunit), and Rbpj (recombination signal binding protein for immunoglobulin kappa J region) (Figure 6B). We used the UCSC joint JASPAR website (http://jaspar2016.genereg.net/) to predict the TF binding sites in the VSIG4 promoter region and combined the results with the mRNA sequencing results. We found that Arnt, Hif1α, and Fosl2 can possibly bind to VSIG4, and subsequently detected the motifs of these TFs (Figure 6C). We then evaluated changes in these TFs in M2 under hypoxic conditions using RT-qPCR and observed a significant difference in Hif1α expression **(**Figure 6D).

In Hif1α-deficient mice, macrophages express lower levels of HIF-regulated genes, such as *VEGF*, than WT macrophages under hypoxic conditions. Interestingly, the exact contribution of Hif1α to the regulation of hypoxic gene expression appears to vary among different cell types 19. Under hypoxic conditions, Hif1α translocates to the nucleus and, together with its partner factor, the basic-helix-loop-helix/PAS protein Arnt, binds to the promoters of target genes 18. To understand whether HIf1α, which is highly expressed in M2 macrophages under hypoxic conditions, plays a role in regulating the transcription of target genes, we investigated whether it can translocate from the cytoplasm to the nucleus. We used western blot and immunofluorescence analyses to detect the relative Hif1α expression in the nucleus and cytoplasm under hypoxic conditions, and the results showed that hypoxia increased Hif1α expression and promoted its translocation from the cytoplasm to the nucleus in M2 macrophages (Figure 6E-6G).

To further determine the transcriptional program regulated by hypoxia, we performed CUT&Tag sequencing on normal and hypoxic M2 macrophages. We combined the CUT&Tag and RNA sequencing analysis and identified 233 consistently upregulated genes, including *VSIG4* and *Hif1α* (Figure 6H). Additionally, the reads in both groups of cells were mainly enriched in the transcription start site (Figure 6I). Further visual analysis using IGV showed a Hif1α binding peak (Peak 6442) at the *VSIG4* promoter (Figure 6J), suggesting that the *VSIG4* promoter region has a Hif1α binding site. Therefore, we mapped Hif1α binding sites to the VSIG4 promoter region according to the binding sites predicted by JASPAR software (Figure 6K). Further analysis using CUT&Tag-qPCR showed a significant increase in Hif1α binding to the *VSIG4* promoter in hypoxic M2 macrophages (Figure 6L). In addition, we investigated the effect of Hif1α on the transcriptional activity of VSIG4 using a dual-luciferase reporter assay for M2 macrophages expressing Hif1α-FLAG, the *VSIG4* promoter pGl3, and pRLTK (thymidine kinase promoter-Renilla luciferase reporter plasmid, used as an internal reference). The results showed that Hif1α upregulated the *VSIG4* promoter activity in M2 macrophages (Figure 6M), suggesting that Hif1α can bind to the VSIG4 promoter region and enhance VSIG4 transcriptional expression.

We further evaluated whether Hif1α affects the transcription and expression of VSIG4 by performing gain- or loss-of-Hif1α-function in M2 macrophages under normoxic and hypoxic conditions. Our results revealed a positive correlation between Hif1α and VSIG4 at both the mRNA and protein levels. Inhibition of Hif1α expression under hypoxic conditions resulted in a concomitant decrease in VSIG4 expression in M2 macrophages (Figure 6N-6W). Collectively, these results suggest that hypoxia leads to increased Hif1α expression in M2 macrophages and promotes its translocation to the nucleus to bind to the promoter region of VSIG4, thereby promoting its transcription and expression.

### Hypoxia Induces Hif1α Expression in M2 Macrophages and Mediates the Expression of VSIG4 to Regulate the Proliferation, Migration, and Phenotypic Transformation of CFs

**Figure 7:**
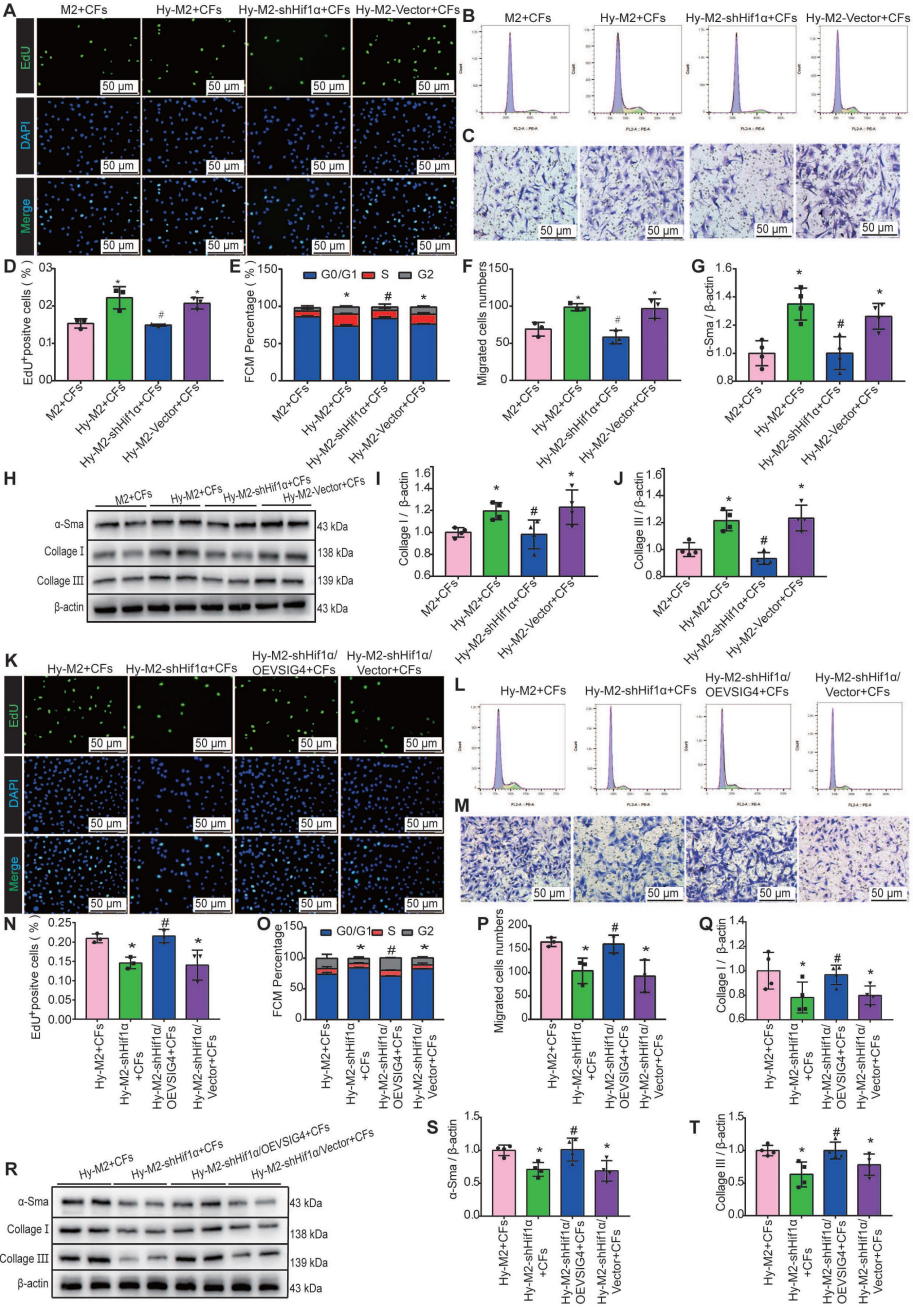
** Hif1α is upregulated in M2 macrophages under hypoxic conditions and mediates VSIG4 expression. A,** Representative EdU fluorescent images (Scale bar, 50 μm). **B,** Representative images of the cell cycle analysis.** C**, Representative pictures of crystalline violet staining for cell migration (Scale bar, 50 μm). **D,** Quantitative analysis of the proportion of EdU-positive cells (n=3, ^*^P<0.05 vs. the M2+CFs group,^ #^*P<*0.05 vs. the Hy-M2+CFs group). **E,** Quantitative analysis of cells in G0/G1, S, and G2 phases in each group (n=3, ^*^P<0.05 vs. the M2+CFs group,^ #^*P<*0.05 vs. the Hy-M2+CFs group). **F,** Quantitative analysis of migrating cells (n=3, ^*^P<0.05 vs. the M2+CFs group, ^#^*P*<0.05 vs. the Hy-M2+CFs group). **G-J,** Representative western blot results of three independent experiments and quantification of α-SMA (smooth muscle actin), collagen I, and collagen III expression in CFs co-cultured with M2 macrophages (n=3, ^*^P<0.05 vs. the M2+CFs group,^ #^*P*<0.05 vs. the Hy-M2+CFs group). **K**, Representative EdU fluorescent images (Scale bar, 50 μm).** L,** Representative images of the cell cycle analysis.** M**, Representative pictures of crystalline violet staining for cell migration (Scale bar, 50 μm). **N,** Quantitative analysis of the proportion of EdU-positive cells (n=3, ^*^P<0.05 vs. the Hy-M2+CFs group,^ #^*P*<0.05 vs. the Hy-M2-shHif1α+CFs group). **O,** Quantitative analysis of cells in the G0/G1, S, and G2 phases in each group (n=3, ^*^P<0.05 vs. the Hy-M2+CFs group,^ #^*P*<0.05 vs. the Hy-M2-shHif1α+CFs group).** P,** Quantitative analysis of migrating cells (n=3, ^*^P<0.05 vs. the Hy-M2+CFs group,^ #^*P*<0.05 vs. the Hy-M2-shHif1α+CFs group). **Q-T,** Representative western blot results of three independent experiments and quantification of α-SMA, collagen I, and collagen III expression in CFs co-cultured with M2 macrophages (n=3, ^*^P<0.05 vs. the Hy-M2+CFs group,^ #^*P*<0.05 vs. the Hy-M2-shHif1α+CFs group).

We further investigated whether Hif1α inhibition can reverse the effects of hypoxia-induced M2 macrophages on CFs. Hif1α-deficient M2 macrophages were pre-cultured under hypoxic conditions and subsequently co-cultured with CFs. The results of EdU, cell cycle, cell migration assays, and western blot assays for α-SMA, collagen I, and collagen III showed that the promotion of proliferation, migration, and phenotypic transformation of CFs by hypoxia-induced M2 macrophages was significantly diminished after Hif1α inhibition (Figure 7A-7J). The expression of TGF-β and IL-10 was significantly decreased in the co-culture system of Hy-M2 with CFs after Hif1α inhibition (Figure S8A and S8B). We further explored whether Hif1α mediates its effects on CFs via VSIG4 by overexpressing VSIG4 in Hif1α-deficient Hy-M2 macrophages. The results showed that co-culturing CFs with Hif1α-deficient Hy-M2 macrophages resulted in diminished pro-proliferation, migration, and phenotypic conversion of CFs, whereas VSIG4 overexpression reversed this effect (Figure 7K-7T).

## Discussion

**Figure 8:**
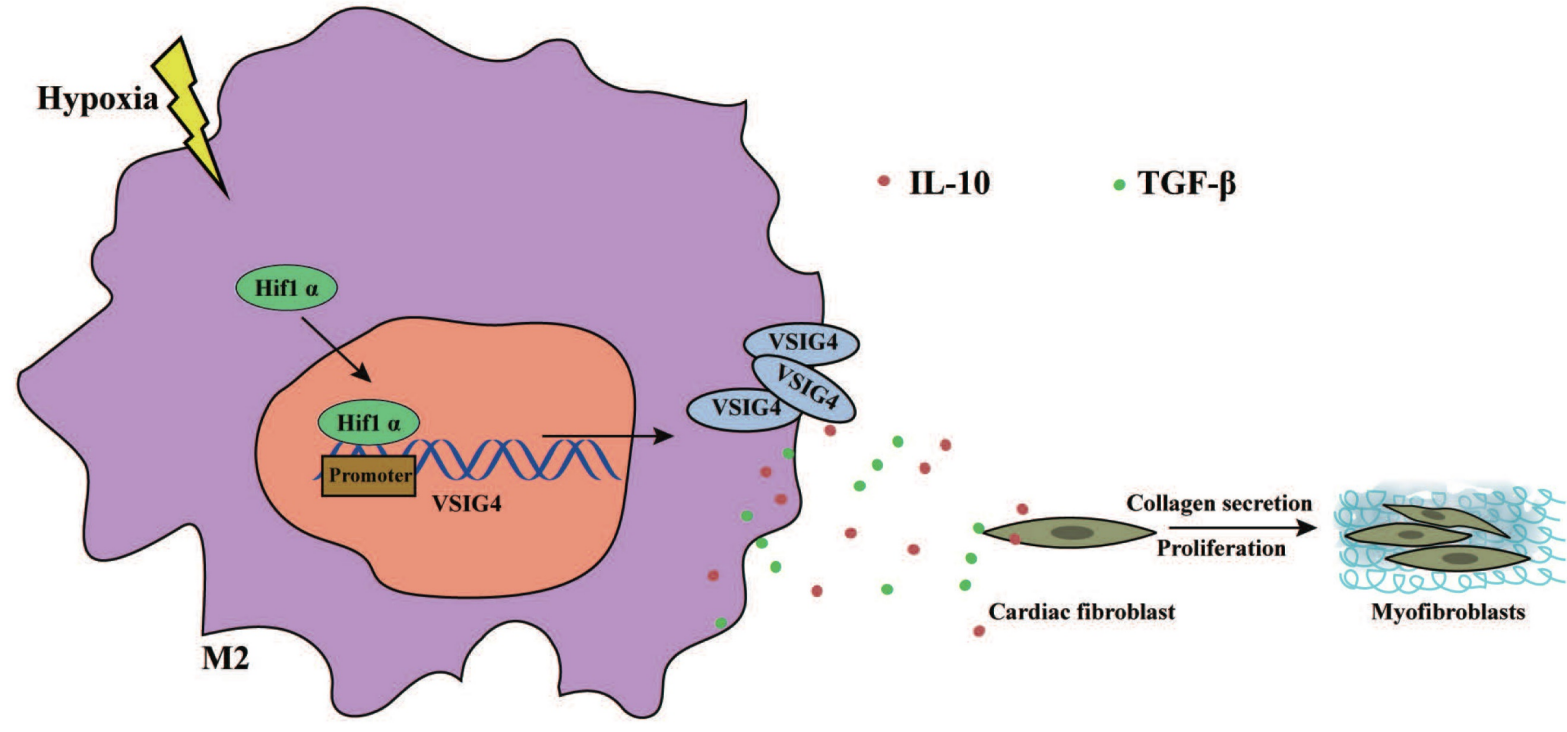
** Graphical Abstract.** Hypoxia promotes the expression and nuclear displacement of Hif1α in M2 macrophages, and subsequently Hif1α binds to the VSIG4 promoter region and mediates VSIG4 expression, thereby promoting the secretion of TGF-β and IL-10, which promote myocardial fibrotic repair after myocardial infarction by regulating the proliferation, migration and phenotypic transformation of CFs.

In this study, we revealed that VSIG4 plays a crucial role in mediating AMI in mice. We showed that myocardium-infiltrating M2 macrophages express VSIG4 and that VSIG4KO leads to impaired scar formation and aggravated left ventricular remodeling, resulting in an increased risk of cardiac rupture and worsened cardiac function in mice with AMI. We also identified two cytokines secreted by VSIG4^+^ M2 cytokines, TGF-β and IL-10, as central coordinators of the wound healing process after AMI. We further revealed that hypoxia mediates the expression of VSIG4 by M2 macrophages, which promotes the secretion of TGF-β and IL-10 and regulates the proliferation, migration, and phenotypic transformation of CFs *in vitro*; therefore, manipulation of this immune axis may assist in the prevention of adverse events in AMI (Figure 8).

AMI in adult mammals results in a massive loss of cardiomyocytes; however, the heart has a limited endogenous regenerative capacity 20. Thus, the infarcted myocardium usually replaces the dead myocardium with matrix-based fibrotic repair, mediating cardiac healing. Immune cells and fibroblasts accumulate in the infarct marginal zone and play a role in remodeling. Infiltrating macrophages regulate the inflammatory response by phagocytosis of dead cells and stromal debris to activate myofibroblast and neointima formation. Activated fibroblasts secrete large amounts of structural extracellular matrix (ECM) proteins that enhance the structural stability of the ventricle while increasing ventricular strength 21. Excessive myocardial injury and poor VR after AMI are associated with death and poor prognosis, and the disruption of the ECM and impaired fibrotic repair may be important mechanisms in myocardial injury and remodeling.

Macrophages play an important role in cardiac repair and revascularization after AMI 17. A recent study has shown that resident macrophages are rapidly depleted after myocardial injury and subsequently rapidly replaced by infiltrate-derived bone marrow mononuclear macrophages 22. Thus, after AMI, the dominant macrophage population in the ischemic region of the myocardial tissue remains in the bone marrow mononuclear macrophage population. Macrophages infiltrating early after myocardial infarction exhibit a typical activation state (M1 type), remove necrotic cell debris by phagocytosis, and promote subsequent tissue repair while releasing pro-inflammatory cytokines to mediate the inflammatory response. Subsequently, they transition to a repair state (M2 type) and reside in the myocardium for a long time, mediating myocardial fibrosis and revascularization through cytokine release 23. The M1/M2 classification initially introduced the concept of innate immune cell integrity. However, recent studies have shown that the macrophage phenotype exhibits greater plasticity than historically assumed, and that the M1 and M2 classifications represent only a continuum of two extreme activation states, as the expression and function of the surface receptors of these macrophages affect their role in regulating inflammation and repair 4-6. Orecchioni *et al.* have shown that inducing the activation of macrophages to express olfactory receptors can significantly exacerbate atherosclerosis in mice 24. Therefore, the expression and function of macrophage surface receptors differ under different pathophysiological conditions.

VSIG4, also known as CRIg or Z39Ig, is a novel member of the B7 superfamily 8. VSIG4 expression is restricted to tissue macrophages, including peritoneal macrophages and liver-hosting Kurtosis cells 25. It negatively regulates inflammatory processes by suppressing T cell function and activating macrophage immunity by binding to C3b/iC3b and mediates transcriptional repression of NLRP3 and Il-1β in macrophages 26. VSIG4 also attenuates NLRP3 via the JAK2-STAT3-A20 pathway and ameliorates neuroinflammation after brain hemorrhage in mice 13. Several studies have reported that VSIG4 expression improves liver fibrosis in hepatic Kupffer cells 27,28. However, the role of VSIG4-positive in cardiac fibrosis after AMI has not been reported yet. In the present study, we screened myocardial macrophages infiltrated at different time points by flow cytometry and performed VSIG4 mRNA sequencing analysis. Our results demonstrated that VSIG4 is highly expressed in macrophages in the late phase after AMI, suggesting that VSIG4 may be critically involved in macrophage-associated inflammation and AMI.

Activated macrophage subpopulations are a major source of cytokines and growth factors that regulate fibrosis by secreting proteases involved in matrix remodeling and producing tissue inhibitors of metalloproteinases 29. Targeted MMP2 knockdown improves survival after myocardial infarction by preventing macrophage infiltration and reducing the rate of left ventricular rupture. In contrast, MMP9 deficiency partially protects against post-infarction rupture and improves left ventricular diastolic and systolic functions 30. In our study, we revealed that VSIG4 inhibits cardiac rupture and remodeling by decreasing the expression of MMP2 and MMP9 and promoting myofibroblast activation and collagen deposition. The protein and mRNA levels of collagen I and III and α-SMA decreased in VSIG4KO mice, indicating a lower level of myofibroblast differentiation in VSIG4KO mice. Activated myofibroblasts migrate into the infarct area, express α-SMA, and produce collagen I and III in abundance, leading to the formation of a collagenous scar that is crucial for the prevention of infarct expansion and left ventricular rupture 31.Therefore, our results suggest that the VSIG4 signaling pathway specifically affects the healing process by modulating the phenotypic transition of fibroblasts to myofibroblasts, potentially mediating myocardial fibrotic repair through the release of cellular and tissue factors that promote ECM remodeling after AMI. Furthermore, using a BMT strategy, we confirmed that VSIG4 expressed on bone marrow-derived M2 macrophages mediates fibrotic repair after AMI, implying that targeting VSIG4 on M2 macrophages may constitute a beneficial therapeutic approach for AMI.

Insights into the interactions between macrophages and fibroblasts may provide greater insights into the progression of VR after myocardial infarction to provide better therapeutic tools. Owing to advances in research, the role of macrophages in post-myocardial infarction can be modulated by targeting specific macrophage surface receptors without affecting the stability of resident macrophages nor impeding the clearance of necrotic material from the infarct site 5,6. In this study, we showed that under *in vitro* conditions, VSIG4 expression was higher in bone marrow-derived M2 macrophages than that in M0 and M1 macrophages. VSIG4 expression in M2 macrophages significantly increased after 24 h of hypoxic culture; however, the morphology and phenotype of M2 macrophages were not affected, and 24 h of hypoxia remarkably promoted M2 macrophage proliferation. Additionally, VSIG4 inhibits macrophage polarization toward the pro-inflammatory M1 type 29, which is important for maintaining the M2 phenotype, by reprogramming mitochondrial pyruvate metabolism. In this study, we found that hypoxic M2 macrophages or M2 macrophages overexpressing VSIG4 co-cultured with cardiac CFs effectively promoted the proliferation, migration, and conversion of CFs into myofibroblasts. However, VSIG4 knockout in M2 macrophages remarkably diminished these M2-mediated effects on CFs after hypoxia. A previous study has suggested that M2 macrophages secrete high levels of IL-10 and TGFβ1 to drive the conversion of α-SMA to protofibroblasts 32, which leads to the conversion of these fibroblasts to myofibroblasts 33. Myofibroblasts have a higher migratory capacity and the ability to secrete ECM components than steady-state fibroblasts 34. Interestingly, in the present study, we also found that IL-10 and TGF-β expression was significantly increased in the co-culture system of hypoxic M2 macrophages or those overexpressing VSIG4 with CFs but was significantly decreased in the co-culture system of VSIG4-deficient M2 macrophages with CFs after hypoxia. Our results indicate that hypoxia induces VSIG4 expression in M2 macrophages, promotes IL-10 and TGF-β secretion by M2 macrophages, and mediates the proliferation, migration, and conversion of CFs to myofibroblasts, thereby mediating tissue repair after AMI and maintaining the structural stability of infarcted myocardium in mice.

In addition, our data extend previous observations and show significantly higher Hif1α expression and nuclear displacement in hypoxia-conditioned M2 macrophages. A combined analysis of mRNA sequencing and CUT&Tag sequencing suggested a Hif1α binding site in the VSIG4 promoter region. Furthermore, Hif1α expression correlated with VSIG4 expression by gain- or loss-of-function of Hif1α under normoxic or hypoxic conditions. These results are consistent with a previous study reporting that HIF-1α is an important TF in the hypoxic microenvironment, is aberrantly highly expressed in hypoxic tissues, and is translocated to the nucleus where it binds to hypoxia response elements to regulate target gene transcription and promote cell survival in the hypoxic microenvironment 35. We observed the functional effects of hypoxic M2 macrophages on CFs by inhibiting Hif1α in M2 macrophages, which showed that hypoxic M2 macrophages with Hif1α inhibition had a diminished ability to promote proliferation, migration, and conversion of CFs to myofibroblasts, and had decreased IL-10 and TGF-β expression levels in the cell co-culture medium. In contrast, VSIG4 overexpression reversed the inhibitory effect of Hif1α.

In conclusion, we demonstrated that VSIG4 contributes scar formation after AMI. VSIG4 orchestrates the myocardial inflammatory response after AMI and promotes TGF-β1 and IL-10. VSIG4 enhances cardiac repair and decreases ventricular rupture and mortality rates through its indirect effects on cardiac fibroblasts in the infarcted area. In the complex environment of the infarcted heart, spatial and temporal regulation of the expression of various cytokines, chemokines, and growth factors may affect macrophages. We hypothesize that the effect of VSIG4+M2 macrophages in regulating fibrotic repair and function after MI, is at least in part, mediated by hypoxia. Future studies are needed to further explore the effect of hypoxic VSIG4+M2 macrophages on cardiac repair after AMI and determine their molecular profile and functional role in the healing process, including their effect on neovascularization after AMI.

## Novelty and Significance

### What Is Known?

Promoting fibrotic repair after myocardial infarction mediates tissue healing and reduces the incidence of cardiac rupture.

Macrophages involved in inflammatory response contribute to the healing process after acute myocardial infarction.

V-set and immunoglobulin domain-containing 4 (VSIG4) is a complement receptor of the immunoglobulin superfamily that is specifically expressed in resting tissue-resident macrophages and participates in a variety of immune responses.

### What New Information Does This Article Contribute?

VSIG4 alleviates wound healing and cardiac remodeling. VSIG4 deficiency deteriorates adverse myocardial remodeling and, in turn, increases ventricular rupture and mortality.

VSIG4 is highly expressed on bone marrow-derived macrophages infiltrating the myocardium in the late post-myocardial infarction period.

In the late post-myocardial infarction phase, VSIG4 increases IL-10 and TGF-β production, which promotes myocardial fibroblast differentiation, migration, and conversion to myofibroblasts in the infarct area, thereby mediating the healing of damaged myocardial tissue.

## Supplementary Material

Supplementary materials and methods, figures and tables.

## Abbreviations

AMI: acute myocardial infarction
α-SMA: α-smooth muscle actin
MMP: matrix metallopeptidase
VR: ventricular remodeling
VSIG4: V-set and immunoglobulin domain-containing 4
CRIg: complement receptor of the immunoglobulin superfamily
Tregs: regulatory T cells
DDR2: discoidin domain receptor 2
Hif1α: hypoxia-inducible factor
Arnt: aryl hydrocarbon receptor nuclear translocator
Fosl: FOS like 1, AP-1 transcription factor subunit
BMT: bone marrow cell transplantation
BMD: bone marrow macrophage depletion
