# Supplementary material for: Hypoxia Induces M2 Macrophages to Express VSIG4 and Mediate Cardiac Fibrosis After Myocardial Infarction
Source: Theranostics. 2023 Apr 2;13(7):2192–209. doi: 10.7150/thno.78736 (PMC10157727; doi:10.7150/thno.78736)
Supplement: Supplementary file 1 — Supplementary materials and methods, figures and tables. [file thnov13p2192s1.pdf]

Hypoxia Induces M2 Macrophages to Express VSIG4 and Mediate Cardiac Fibrosis After Myocardial Infarction

Yan Wang[1,2#], Yu Zhang[1#], Jiao Li[1#], Chaofu Li[1], Ranzun Zhao[1], Changyin Shen[1], Weiwei Liu[1], Jidong Rong[1], Zhenglong Wang[1*], Junbo Ge[3*], and Bei Shi[1,2*]

[1]Department of Cardiology, Affiliated Hospital of Zunyi Medical University, Zunyi 563000, China

[2]Soochow University, Suzhou, China

[3]Department of Cardiology, Zhongshan Hospital, Fudan University, Shanghai Institute of Cardiovascular Diseases, Shanghai, China

*Corresponding author:

Name: Bei Shi, Junbo Ge and Zhenglong Wang

No. 149, Dalian Road, Huichuan District, Zunyi City, Guizhou, China

Email address: **shib@zmu.edu.cn**

[#] These authors contributed to this work equally.

Supplemental Materials and Methods

In Vivo Experimental Grouping

To examine the functional role of VSIG4, VSIG4 knockout (VSIG4KO) mice were divided into two groups (n = 4–6): sham (VSIG4KO + sham) and acute myocardial infarction (VSIG4KO +AMI) groups. In this set of experiments, C57BL/6 mice were also assigned to the sham (WT + sham) and AMI groups (WT + AMI) as wild-type (WT) controls. Bone marrow transplantations (BMTs) were performed between WT and VSIG4KO mice. Six weeks after BMT, mice were subjected to AMI. The following groups of mice were studied: WT→WT, VSIG4KO→WT, WT→VSIG4KO, and VSIG4KO→VSIG4KO mice.

Establishment of the AMI Mouse Model

Adult mice were anesthetized by 2% isoflurane inhalation. After disinfection, the skin was cut between the third and fourth ribs on the left edge of the sternum, and the pectoralis major and external intercostal muscles were blunt-separated. The intercostal space was pierced between the third and fourth ribs using a pair of curved hemostatic forceps. Immediately thereafter, the heart was squeezed out using the left thumb and index finger. The left anterior descending artery (LAD) was ligated using 8.0/5.0 needle sutures. Similar procedures were performed without ligation in the sham-operated group.

Echocardiography

Heart function was examined using echocardiography with a Vevo 2100

ultra-high-resolution small-animal imaging system (VisualSonics, Toronto, ON, Canada). Four weeks after AMI, mice anesthetized with a mixture of 1.5% isoflurane and oxygen (1 L/min) were subjected to echocardiographic studies. Left ventricular ejection fraction (LVEF) and fractional shortening (FS) were calculated using the corresponding formulae, as described in a previous study [1]. Echocardiographic acquisition and analysis were performed by a technician who was blinded to the groups.

Histological Examinations

Fresh heart tissues were fixed in 4% paraformaldehyde for 24 h, embedded in paraffin, and sectioned with 4 μm. The sections were sequentially deparaffinized with xylene and gradient ethanol, and Masson's trichrome staining kit and hematoxylin and eosin (HE) staining kit (Solarbio) were used to detect fibrotic areas according to the manufacturer's instructions. The area of myocardial infarction fibrosis was measured using ImageJ software.

Immunofluorescence Analysis

Fresh heart tissues were sliced using an optimal cutting temperature (OCT) compound. Tissue sections and cultured cells were fixed in 4% paraformaldehyde for 30 min and permeabilized with 1% Triton X-100 in phosphate-buffered saline (PBS) for 10 min. The samples were then incubated with goat serum at 25 °C for 1 h. Afterwards, the samples were incubated overnight at 4 °C with the following primary antibodies diluted with goat serum: anti-CD206, anti-VSIG4, and anti-collagen III.

The next day, the samples were stained with fluorescent secondary antibodies at room temperature for one hour. Nuclei were stained with 4,6-diamino-2-phenylindole (DAPI; Sigma-Aldrich, St. Louis, MO, USA) for 10 min. Images were obtained using a fluorescence microscope (Olympus, Tokyo, Japan).

Bone Marrow Cell Preparation and BMT

To harvest bone marrow cells, femurs and tibias were obtained from 8-week-old male WT or VSIG4KO mice. The bone marrow was flushed out with a 24-gauge syringe by flushing with a sterile medium (Dulbecco's modified Eagle's medium containing 10 U/mL heparin, 50 U/mL penicillin, and 50 μg/mL streptomycin), and a single-cell suspension in PBS was obtained by filtering the suspension through a 100-μm nylon mesh after red blood cell lysis. A total of 5×10^6 bone marrow cells were transferred intravenously into lethally irradiated (9 Gy from an X-ray source) 6–8-week-old male WT or VSIG4KO mice [2]. In the BMD group, mice were irradiated with lethal radiation (9 GY) to remove bone marrow and peripheral mononuclear macrophages prior to AMI, followed by transfering of mononuclear macrophage-free PBS.To evaluate bone marrow chimerism, after BMT, $CD45.1^+$ C57/b6 and VSIG4KO ($CD45.2^+$), mice were used to perform BMT under the same conditions as described above. The mice were allowed to rest for 6 weeks, and the success of BMT was confirmed by flow cytometric analysis of the peripheral blood.

Flow Cytometric Analysis

Single-cell suspensions were prepared as previously described with some

modifications [3]. Briefly, mice were deeply anesthetized and intracardially perfused with 40 mL of ice-cold PBS to eliminate blood cells. The hearts were dissected, minced with fine scissors, and enzymatically digested in Hanks' Balanced Salt Solution (HBSS, H9269, Sigma-Aldrich) with a cocktail of 1 mg/mL type II collagenase (Worthington Biochemical Corporation, Lakewood, NJ, USA), 100 U/mL elastase (Worthington Biochemical Corporation), and 100 U/mL DNase I (Sigma-Aldrich) for 1.5 h at 37 °C with gentle agitation. Following digestion, tissue samples were triturated and passed through a 70-μm cell strainer (BD Biosciences, Franklin Lakes, NJ, USA). Single-cell suspensions were centrifuged (15 min, 500 × *g*, 4 °C). The cells obtained were enriched. Then, the isolated cells were counted after erythrocyte lysis and washed with RPMI-1640 cell culture medium for further analysis. To block the nonspecific binding of antibodies to Fcγ receptors, the isolated cells were incubated first with anti-CD16/32 antibody (2.4G2, BD Biosciences, Franklin Lakes, NJ, USA) at 4 °C for 5 min. Subsequently, the cells were incubated with a mixture of antibodies at 4 °C for 20 min for cytometric analysis. The antibodies used in this study are listed in Supplementary Table 1. The results were expressed as the percentage or cell number per microgram of tissue. Flow cytometric analysis and cell sorting were performed using an LSRFORTESSA and FACSAria instrument (BD Biosciences) and analyzed using FlowJo software (Tree Star).

Polarization and Hypoxia Induction in Bone Marrow-Derived M2 Macrophages

Bone marrow-derived macrophages were flushed out from the femurs and tibias

of 6-week-old mice and differentiated in complete Dulbecco's modified Eagle's medium (DMEM)/F12 medium supplemented with 50 ng/mL macrophage colony-stimulating factor (MCSF) [4]. On day 7, the macrophages were treated with interleukin (IL-4) for 24 h to generate M2 macrophages. To establish a hypoxia model of M2 macrophages, M2 macrophages were then incubated in a hypoxia incubator for 24 h. Transmission electron microscopy (Hitachi H7500; Tokyo, Japan) and flow cytometry were used to identify M2 polarization.

Preparation of Cardiac Fibroblasts (CFs)

Primary cultures of mouse CFs were prepared as previously described [20]. Briefly, mouse hearts were removed under aseptic conditions, and the residual blood in the heart chambers was repeatedly rinsed off with pre-chilled PBS, followed by cutting the ventricles into tissue pieces of approximately 1 mm^3 with ophthalmic scissors. The tissue pieces were transferred into sterile bottles with one sterilized magnetic rotor and tissue digestion solution (with 0.125% trypsin; Invitrogen, Waltham, MA, USA), placed in a 37 °C water bath and stirred for 40 min until the tissue pieces were flocculent. A complete medium (DMEM supplemented with 10% fetal calf serum) was then added to terminate the digestion. The digest was then filtered through a cell filter (400 mesh), and the filtrate was centrifuged at 600 × *g* for 5 min. The precipitate was resuspended in a complete medium and incubated in 25-cm^2 culture flasks in a 5% CO_2 incubator, and the cells were treated with pancreatic enzymes (containing 0.25% trypsin and 0.02% EDTA) and subcultured. CFs from passages 2 to 4 were assessed by labeling with anti-vimentin antibodies and

discoidin domain receptor 2 (Sigma-Aldrich). The serum-containing medium from cultured cells was replaced with a serum-free medium for 24 h to synchronize the cell cycle for subsequent experiments.

Adenoviral Vector Transfection

Adenoviral vectors carrying enhanced green fluorescent protein (EGFP) and VSIG4 overexpression or shRNA were synthesized by Hanbio (Shanghai, China). Forty-eight hours after transfecting M2 macrophages at 50 MOI (multiplicity of infection), EGFP (enhanced green fluorescent protein) expression was detected by immunofluorescence and real-time quantitative PCR (qRT-PCR) or western blotting was used to evaluate gene expression.

Cell Co-Culture

To verify that hypoxia induces VSIG4 expression in M2 macrophages to regulate CFs, M2 macrophages were cultured under hypoxic conditions for 24 h and VSIG4 was overexpressed or inhibited using the transfection method described above. These M2 macrophages were co-cultured with CFs [4]. The two cell types in the chambers were co-cultured for 24 h for further experiments.

EDU Proliferation Assay

Cell proliferation was detected using Cell-light EDU Apollo 567 *in vitro* kit (RiboBio Co. Ltd., Guangzhou, China) according to the manufacturer's instructions. First, cells were incubated in EdU medium for 2 h and fixed in 4% paraformaldehyde at room temperature for 30 min. Then they were stained with Apollo staining solution

for 30 min, and their nuclei were stained with 4',6-diamidino-2-phenylindole (DAPI). Images were captured using a fluorescence microscope (Olympus).

Cell Cycle Assay

Cells were collected and fixed in 70% cold ethanol for 24 h. They were stained with propidium iodide (PI) staining solution at 37 °C for 30 min, and the samples were analyzed using flow cytometry (Guava; MilliporeSigma; Burlington, MA, USA).

Transwell Migration Assay

One hundred microliters of CF suspension (1×10^4 cells) were added to the Transwell chamber (3422, Beyotime Biotechnology), and 600 µL of a complete medium was added to the lower chamber. Transwell inserts were incubated in an incubator at 37.5 ° C under 5% CO_2 conditions for 24 h. Non-migrated cells in the upper chamber were gently wiped off with cotton swabs. The chamber was washed with PBS, fixed with 4% paraformaldehyde for 30 min, and stained with 1% crystal violet for 15 min. Five random fields of view were photographed and counted using an inverted microscope.

qRT-PCR Assays

Total RNA was isolated from tissues or cells using TRIzol reagent (Takara, Dalian, China), and RNA was reverse transcribed into cDNA using the PrimeScript RT Kit (Takara) according to the manufacturer's instructions. The amplification was performed under the following conditions using TB Green™ premix Ex Taq™ II Reagent Kits (Takara, Dalian, China): pre-denaturation (1 repeat), 95 °C × 30 s; PCR reaction (40 repeats), 95 °C × 3 s (denaturation), 60 °C × 30 s (annealing, extension);

and 95 °C × 5 s, 60 °C × 1 min, 95 °C × 0.11 °C/s (melting), and 50 °C × 30 s (cooling). Relative gene expression levels were quantified using the $2^{-\Delta\Delta CT}$ method.

Nuclear Extraction and Western Blotting

Normoxic or hypoxic M2 macrophages were washed thrice with chilled PBS and then harvested by scraping with an equal volume of PBS, followed by centrifugation at 1000 × *g* for 5 min at 48 °C. Cells were lysed in PBS containing 0.5% Igapal, protease inhibitor cocktail, and phosphatase inhibitor cocktail (Sigma-Aldrich). Cells were mixed thoroughly with the lysis buffer on ice for approximately 5 min, and aliquots from the total lysate were collected and resuspended by adding pre-chilled CER I solution and shaking. The cells were resuspended and placed on ice for 10 min. Subsequently, pre-chilled CER II solution was added, shaken vigorously, mixed, and left on ice for 10 min. The supernatant was collected as cytoplasmic protein by centrifugation at 12,000 × *g* for 5 min at 4 °C. The remaining precipitate was added to the pre-chilled NER solution, placed on ice, shaken vigorously every 10 min for a total of four times, then centrifuged at 12,000 × *g* for 5 min at 4 °C. The supernatant was collected as cytosolic proteins.

Proteins were extracted, quantitated, separated by 12% SDS-PAGE gels, and transferred to PVDF membranes (MilliporeSigma). The membranes were blocked with 5% skim milk and incubated with primary antibodies at 4 °C overnight. They were then incubated with a horseradish peroxidase (HRP)-conjugated secondary antibody at room temperature for 1 h. Protein signals were assessed using enhanced chemiluminescence (ECL; Beyotime Biotechnology) reagent on a ChemiDoc MP

system, and the grayscale value of the target band was analyzed using ImageJ software.

RNA-Seq

RNA-seq of M2 and hypoxic-M2 (Hy-M2) (n=3 per group) was performed using Novogene and Illumina X TEN. Six GB of clean data per sample were collected for RNA-seq. Hg38 assembly was used for read alignment and gene annotation was performed using Ensembl gene annotation version 90.

High-Throughput Cleavage Under Targets and Tagmentation (CUT&Tag)

The CUT&Tag assay was performed as described previously with modifications [5]. Briefly, 100,000 cells were washed twice gently with wash buffer (20 mM HEPES, pH 7.5, 150 mM NaCl, 0.5 mM spermidine, and 1 × protease inhibitor cocktail) at room temperature. Ten microliters of Concanavalin A-coated magnetic beads (Bangs Laboratories Inc., Fishers, IN, USA) were added to each sample and incubated at room temperature for 10 min. The supernatant was removed and bead-bound cells were resuspended with dig wash buffer (20 mM HEPES pH 7.5; 150 mM NaCl; 0.5 mM Spermidine; 1 × Protease inhibitor cocktail; 0.05% Digitonin; and 2 mM EDTA) and a 1:50 dilution of primary antibody or IgG control antibody (normal mouse IgG: MilliporeSigma, cat. no. 12–371) and incubated on a rotating platform overnight at 4 °C. The primary antibody was then removed using a magnetic stirrer. The secondary antibody (rabbit anti-mouse IgG H&L; ab6709; Abcam, Cambridge, UK) was diluted

(1:100) in dig wash buffer and cells were incubated at room temperature for 60 min, and then washed using the magnet stand 2–3 times in dig wash buffer. A 1:100 dilution of pA-Tn5 adapter complex was prepared in dig-med buffer (0.01% Digitonin; 20 mM HEPES pH 7.5; 300 mM NaCl; 0.5 mM Spermidine; and 1 × Protease inhibitor cocktail) and incubated with cells at room temperature for 1 h. The cells were washed 2–3 times for 5 min in 1 mL of Dig-med buffer, resuspended in tagmentation buffer (10 mM $MgCl_2$ in Dig-med Buffer), and incubated at 37 °C for 1 h. DNA was purified by phenol-chloroform-isoamyl alcohol extraction and ethanol precipitation. To amplify the libraries, 21 μL of the DNA solution was mixed with 2 μL of universal i5 and uniquely barcoded i7 primers. A volume of 25 μL NEBNext High-Fidelity 2 × PCR Master mix was added and mixed. The sample was placed in a thermocycler with a heated lid using the following cycling conditions: 72 °C for 5 min (gap filling); 98 °C for 30 s; 14 cycles of 98 °C for 10 s and 63 °C for 30 s; final extension at 72 °C for 1 min and at 8 °C. Library clean-up was performed using XP beads (Beckman Coulter, Brea, CA, USA).

Luciferase Reporter Assay

VSIG4 promoter activity was measured as previously described [39]. HEK293 cells were plated at a density of 1 × 10^5 cells/well in 24-well plates for 24 h and then transfected with different plasmids (Supplementary Table 3) using Lipofectamine 3000 reagent (Invitrogen), following the manufacturer's instructions. For solution A, 100 μL of DMEM was mixed with 1.2 μg of the target plasmid (PRL: 0.2 μg, promoter: 0.4 μg, transcription factor: 0.6 μg) at room temperature. For solution B, 10

μL of DMEM was mixed with 2 μL of transfection reagent (transfection reagent is Hanheng biological product, concentration is 0.8 mg/ml). Solutions A and B were mixed and incubated at room temperature for 20 min. The cell culture medium was discarded and replaced with fresh medium, after which the transfection mixture was added; the cells were incubated at 37 °C under 5% CO_2 conditions. The 24-well plates were removed from the incubator and incubated until the cells equilibrated to room temperature. Passive Lysis Buffer reagent (500 μL) was added to each well. Cells were dispersed by pipetting with a pipette gun, incubated for 10 min at room temperature, then centrifuged at 500 g at 4 °C for 10 min. Then, 40 μL of LAR I was added to 96-well plates, and 50 μL of LAR II was added to each well. Luciferase activity was recorded using an enzyme meter. Next, 50 μL of Stop & Glo® Reagents were added to each well. The transfected cells were assayed for Firefly and *Renilla* luciferase activities in a luminometer by the Dual-Luciferase Reporter Assay System (Beyotime), according to the manufacturer's instructions. The luciferase intensities of each sample were normalized against the pRL-TK levels, and the relative light unit intensity was calculated as the ratio of firefly luciferase to *Renilla* luciferase. All experiments were performed in triplicate.

ELISA

The supernatants of the co-culture system were collected and TGFβ and IL-10 expression levels were analyzed. TGFβ concentration was determined using a commercially available mouse TGFβ Quantikine ELISA Kit, and IL-10 concentration was measured using a Mouse IL-10 Quantikine ELISA Kit, according to the

manufacturer's instructions. Following the addition of the stop solution, absorbance was measured at 450 nm (ELx800, BioTek Instruments).

Supplementary Table 1. The list of the antibodies and kits used in this study.

**Antibody specificity**	**Company**	**Cat. No.**	**Application**	**Dilution**
**Mouse antibodies for fluorescence-activated cell sorting (FACS)**				
BV510™ anti-mouse CD45	BioLegend	103138	FACS	1:400
FITC anti-mouse/human CD11b	BioLegend	101205	FACS	1:400
PE/Cy7 anti-mouse F4/80	BioLegend	123114	FACS	1:400
PerCP/Cy5.5 anti-mouse CD206	BioLegend	141716	FACS	1:400
PerCP/Cy5.5 anti-mouse CD11b	BioLegend	101228	FACS	1:400
FITC anti-mouse F4/80	BioLegend	123107	FACS	1:400
Rabbit Anti-VSIG4/PE Conjugated antibody	Bioss	Bs-0479R-PE	FACS	1:400
Rabbit Anti-CD206, Alexa Fluor 750 conjugated antibody	Bioss	bs-4727R -AF750	FACS	1:400
BV 510™ anti-mouse CD	BioLegend	110741	FACS	1:400

45.1				
FITC anti-mouse CD45.2	BioLegend	109805	FACS	1:400
**Mouse antibodies for western blot**				
GAPDH	ProteinTech	60004-1-1g	WB	1:3000
Tubulin	Sigma-Aldrich	T9026	WB	1:3000
α-SMA	Abcam	ab28052	WB	1:1000
Collagen I	Santa Cruz	SC8784	WB	1:1000
Collagen III	Abcam	ab7778	WB	1:1000
MMP2	Abcam	ab92536	WB	1:1000
MMP9	Abcam	ab283575	WB	1:1000
**Mouse antibodies for immunofluorescence analysis**				
α-SMA	Abcam	ab28052	IF	1:200
Collagen I	Abcam	Ab270993	IF	1:200
Collagen III	Abcam	ab7778	IF	1:200
F4/80	AbD Serotec	MCA497GA	IF	1:200
CD206	Proteintech	Cat NO.60145-1-Ig	IF	1:200
VSIG4	Abcam	Ab252933	IF	1:200
ProLong® Gold Antifade Reagent with DAPI	Invitrogen	P36931	IF	
**Other kits**				

TTC	Sigma-Aldrich	T8877-5G		1%
Crystal Violet Staining Solution	Beyotime Biotechnology	C0121	Cell migration experiment	
Cell cycle kit	4A BIOTECH	FXP0211	FACS	
EdU kit	Ribobio	C10310	IF	
Masson trichrome staining kit	Solarbio	G1340		
**Mouse ELISA kit**				
TGF-β ELISA	R&D Systems	PDB100C		
Il-10 ELISA	R&D Systems	PM1000B		

MMP; matrix metallopeptidase; α-SMA, smooth muscle α; TGF-β, tumor growth factor β; ELISA, enzyme-linked immunosorbent assay

Supplementary Table 2. The list of primer sequences used in this study.

Gene	Primer sequence
VSIG4 Forward	5-GGGTGTTTGCCAGGAAG-3
VSIG4 Reverse	5-GTTCATCAGTTGCGATGGT-3
Collagen I Forward	5-CAGAGGCGAAGGCAACA-3
Collagen I Reverse	5-GTCCAAGGGAGCCACATC-3
Collagen III Forward	5-AGAACCTGGCCGACATC-3
Collagen III Reverse	5-TGGACTTCCGGGCATAC-3

α-SMA Forward	5-GCCCAGAGCAAGAGAGG-3
α-SMA Reverse	5-TGTCAGCAGTGTCGGATG-3
Hif1α Forward	5-TGAACCCATTCCTCATCC-3
Hif1α Reverse	5-CGGCCCAAAAGTTCTTC-3
Arnt Forward	5-AGACCACAGGACAGTTCCA-3
Arnt Reverse	5-GGCTAGGTGCTTGTGCTT-3
Fosl2 Forward	5-TGCCTTGGCTCTAACTCTG-3
Fosl2 Reverse	5-TTGTGCTTCTAGGGCTCTG-3
Promoter region of VSIG4-1 Sense	5-TGTCACTTCCATGGACTAGGGC-3
Promoter region of VSIG4-1 Anti-sense	5-TCCCTGGGTCTGACCTCAGT-3
Promoter region of VSIG4-2 Sense	5-CCTGAGTGCAAATTTCCCCTTTG-3
Promoter region of VSIG4-2 Anti-sense	5-GCCTTGTACTTGCTCAGTATGCA-3

α-SMA, smooth muscle actin; HIF1, α, hypoxia-inducible factor; Arnt, aryl hydrocarbon receptor nuclear translocator; Fosl, FOS like 1, AP-1 transcription factor subunit; VSIG4, V-set and immunoglobulin domain-containing 4

Supplementary Table 3. Grouping of promoter transcription factor activity assays.

Groups	Promoter	Transcription factors
1	pGL3-basic-NC	pcDNA3.1-NC
2	m-vsig4-pro-wt	pcDNA3.1-NC
3	m-vsig4-pro-mut	pcDNA3.1-NC
4	pGL3-basic-NC	PC-m-Hif1α

5	m-vsig4-pro-wt	PC-m-Hif1α
6	m-vsig4-pro-mut	PC-m-Hif1α

NC, Negative control; mut, mutated; Hif1α, hypoxia-inducible factor.

References

1. Long X P,Qiu Z M, Shi B et al. CircERBB2IP promotes post-infarction revascularization via the miR-145a-5p/Smad5 axis. Mol. Ther. Nucleic Acids 28, 573-586 (2022).10.1016/j.omtn.2022.04.006.PMID：35592503
2. Hu Y, Zhang H, Lu Y, Chen Q, et al.Class A scavenger receptor attenuates myocardial infarction-induced cardiomyocyte necrosis through suppressing M1 macrophage subset polarization. Basic Res. Cardiol. 106, 1311-1328 (2011).10.1007/s00395-011-0204-x .PMID：21769674
3. Yan X, Anzai A, Katsumata Y, et al., Temporal dynamics of cardiac immune cell accumulation following acute myocardial infarction. J. Mol. Cell. Cardiol. 62, 24-35 (2013).10.1016/j.yjmcc.2013.04.023.PMID：23644221
4. Wang Y, Li C, Shi B, et al. CircUbe3a from M2 macrophage-derived small extracellular vesicles mediates myocardial fibrosis after acute myocardial infarction. Theranostics 11, 6315-6333 (2021). 10.7150/thno.52843 .PMID：33995660
5. Kaya-Okur HS, Wu SJ, Codomo CA.,et al., CUT&Tag for efficient epigenomic profiling of small samples and single cells. Nat. Commun. 10, 1930 (2019).10.1038/s41467-019-09982-5 .PMID：31036827

Supplementary Figures

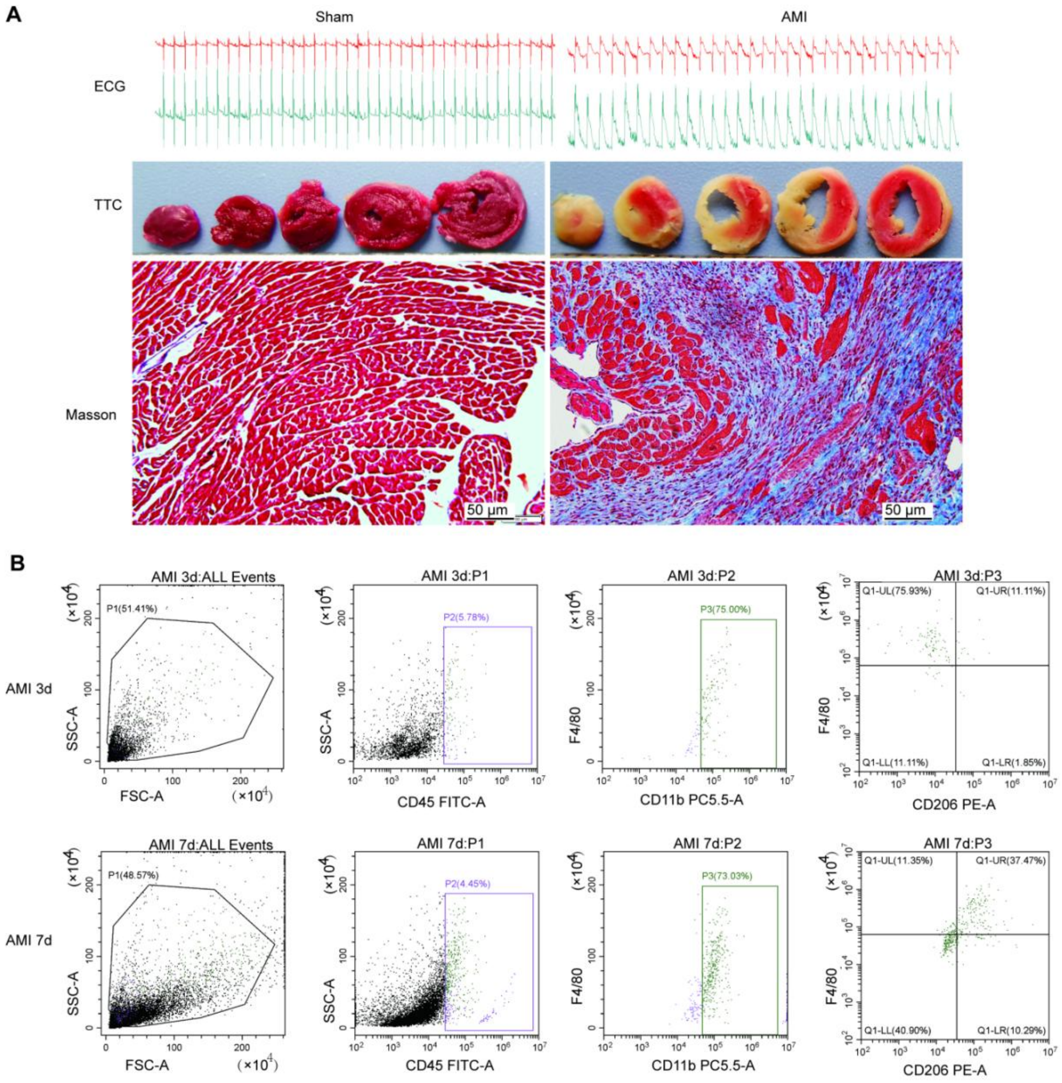

Supplementary Figure 1. Acute myocardial infarction model preparation and macrophage sorting strategy. A, Validation of acute myocardial infarction (AMI) model by Electrocardiograph (ECG), 2, 3, 5-triphenyltetrazolium chloride (TTC) staining, and Masson staining (Scale bar, 50 μm). **B,** Flow cytometric analysis of $CD11b^{+}F4/80^{+}$ macrophages in the heart on days 3 and 7 after AMI.

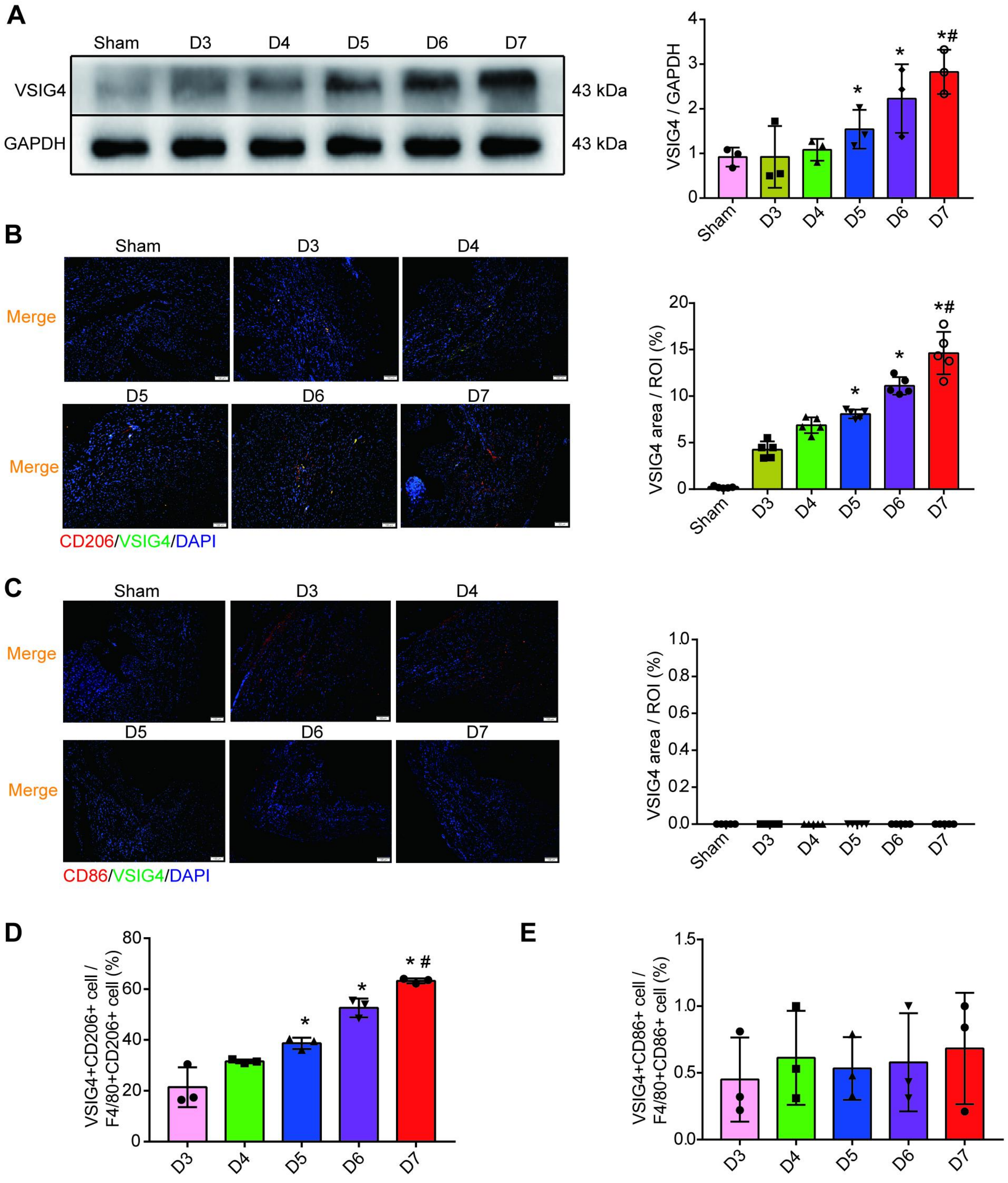

Supplementary Figure 2. the expression of VISG4 on M1 or M2 macrophages.A, VSIG4 protein expression at different time points in the heart (n=3, $^{*}P$<0.05 vs. the sham group, $^{\#}P$<0.05 vs. the D6 group).**B,**left**:**Representative dual-immunofluorescence staining for VSIG4 and CD206 in heart at different time points of AMI (Scale bar, 50 μm) .Right:A histogram showing VSIG4 fluorescence intensity (n=5, $^{*}P$<0.05 vs. the sham group, $^{\#}P$<0.05 vs. the D6

group).**C,**left**:**Representative dual-immunofluorescence staining for VSIG4 and CD 86 in heart at different time points of AMI (Scale bar, 50 μm) .Right:A histogram showing VSIG4 fluorescence intensity.**D.**Temporal dynamics of VSIG4$^+$CD206$^+$macrophage count in infarct area(n=3, $^*P$<0.05 vs. the sham group, $^\#P$<0.05 vs. the D6 group). **E.**Temporal dynamics of VSIG4$^+$CD86$^+$macrophage count in infarct area (n=3, $^*P$<0.05 vs. the sham group, $^\#P$<0.05 vs. the D6 group).

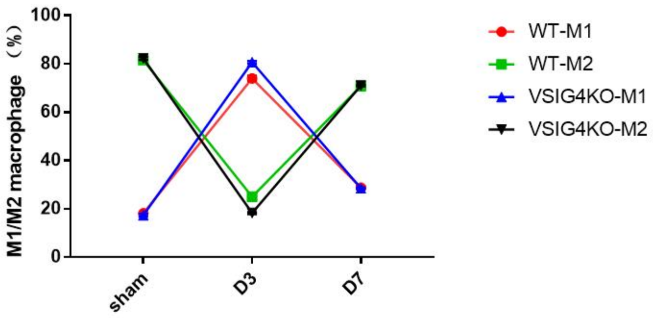

Supplementary Figure 3. M1 and M2 Macrophage infiltration in VSIG4KO and WT mice after AMI.

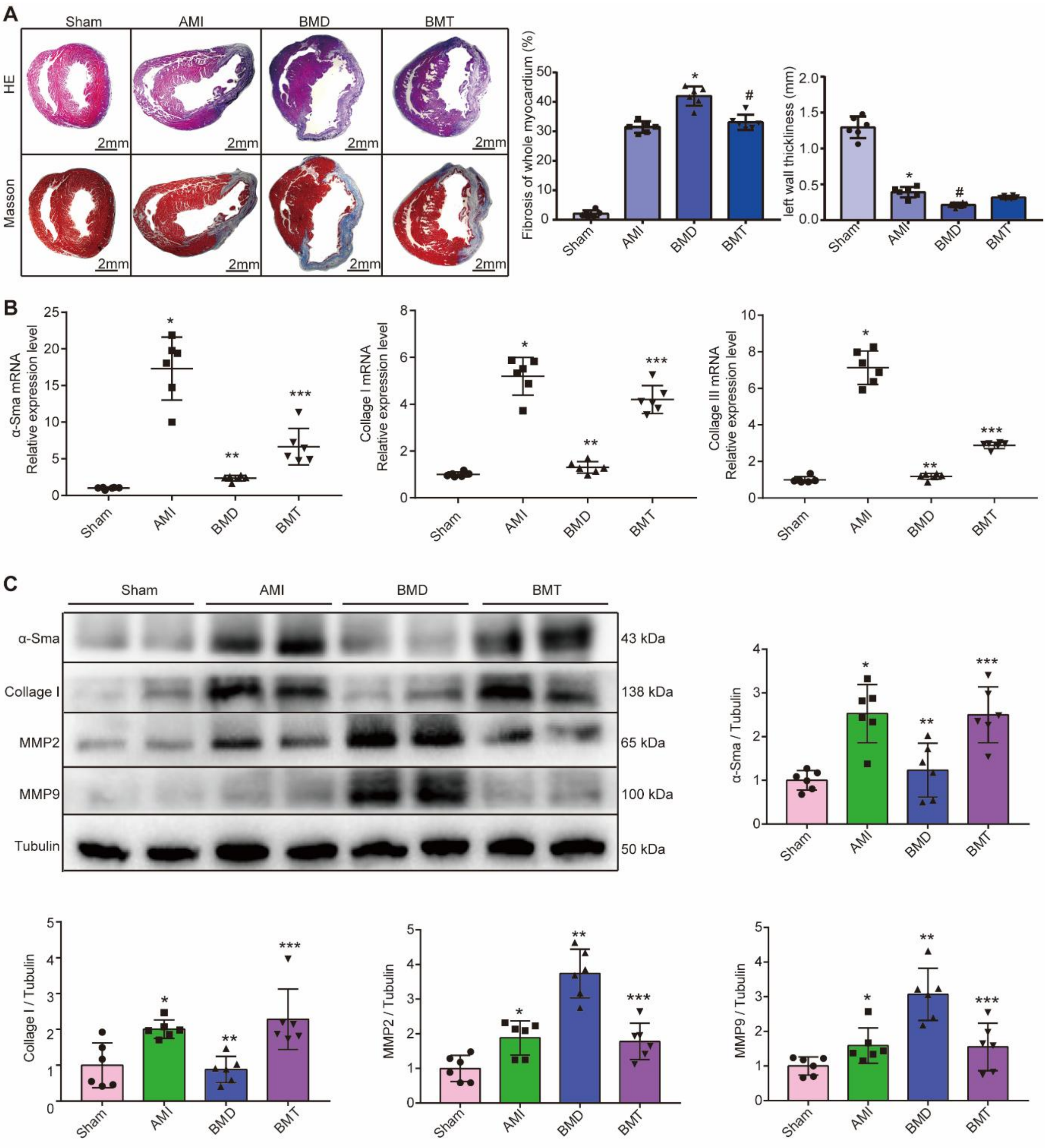

Supplementary Figure 4. The effects of bone marrow macrophages on the repair of cardiac fibrosis after acute myocardial infarction (AMI). A, left: Representative Masson's trichrome staining and hematoxylin and eosin (HE) staining of cardiac tissues obtained from BMD (bone marrow depletion) or BMT (bone marrow transplant) mice 28 days after AMI or sham operation. Right: Quantitative analysis of infarct size and wall thickness 28 days after AMI in sham, AMI, BMD, and BMT

mice. **B,** Gene expression levels of collagen I, collagen III, and α-SMA in different groups. **C,** Protein expression levels of collagen I, α-SMA, and MMP2 7 days after AMI in the scar tissue isolated from the sham, AMI, BMD, and BMT groups (n=6, $^{*}P < 0.05$ vs. AMI group, $^{\#}P < 0.05$ vs. BMD group).

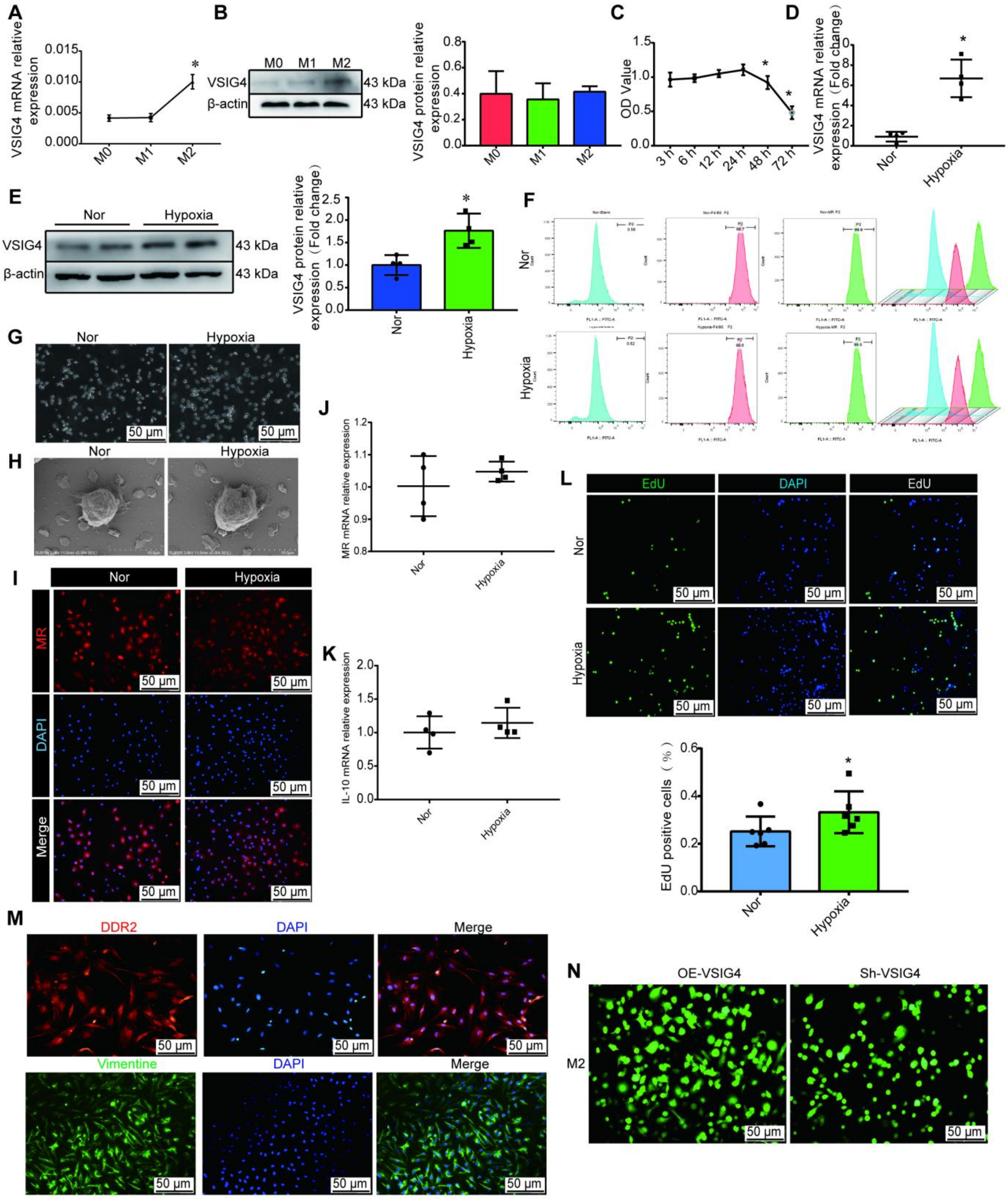

Supplementary Figure 5. Effects of hypoxia on the phenotype of M2

macrophages. A, Detection of VSIG4 mRNA expression in various macrophage types (n=3, $^{*}P < 0.05$, vs. M0 or M1 group). **B**, Representative western blot images of VSIG4 expression in M0, M1, and M2 macrophages. Quantitative analysis of VSIG4 in M0, M1, and M2 macrophages (n=3). **C.** Cell counting kit 8 assay (CCK8) showing the effect of hypoxia on the viability of M2 macrophages (n=3, $^{*}P < 0.05$ at 3, 6, 12, and 24 h). **D,** Real-time quantitative polymerase chain reaction (RT-qPCR) analysis showing VSIG4 mRNA expression levels in normoxic (nor) and hypoxic M2 macrophages (n = 4, $^{*}P < 0.05$, vs. Nor group). **E,** Western blot analysis showing VSIG4 expression in normoxic and hypoxic M2 macrophages (n = 4, $^{*}P < 0.05$, vs. Nor group). **F,** Flow cytometry analysis showing the expression of F4/80 and (Mannose receptor) MR in normoxic and hypoxic M2 macrophages. **G,** Observation of M2 macrophage morphology under normoxic or hypoxic conditions under a light microscope (Scale bar, 50 μm). **H,** Observation of normoxic M2 macrophage morphology using a transmission electron microscope (TEM). **I,** Immunofluorescence analysis of MR (red) expression in normoxic and hypoxic M2 macrophages (Scale bar, 50 μm). **J, K,** RT- qPCR analysis showing the relative expression of the M2 macrophage surface markers MR and interleukin (IL)-10 (n=4). **L,** EdU assay showing a positive association between hypoxia and M2 proliferation (n = 6, $^{*}P <$ 0.05, vs. Nor group). **M,** Immunofluorescence analysis for discoidin domain receptor 2 (DDR2) (red) and vimentin (green) in cardiac fibroblasts (CFs) (Scale bar, 50 nm). **N**, Immunofluorescence analysis of enhanced green fluorescent protein (EGFP) in M2 macrophages overexpressing VSIG4 by adenovirus transfection for 48 h (Scale bar,

50 μm).

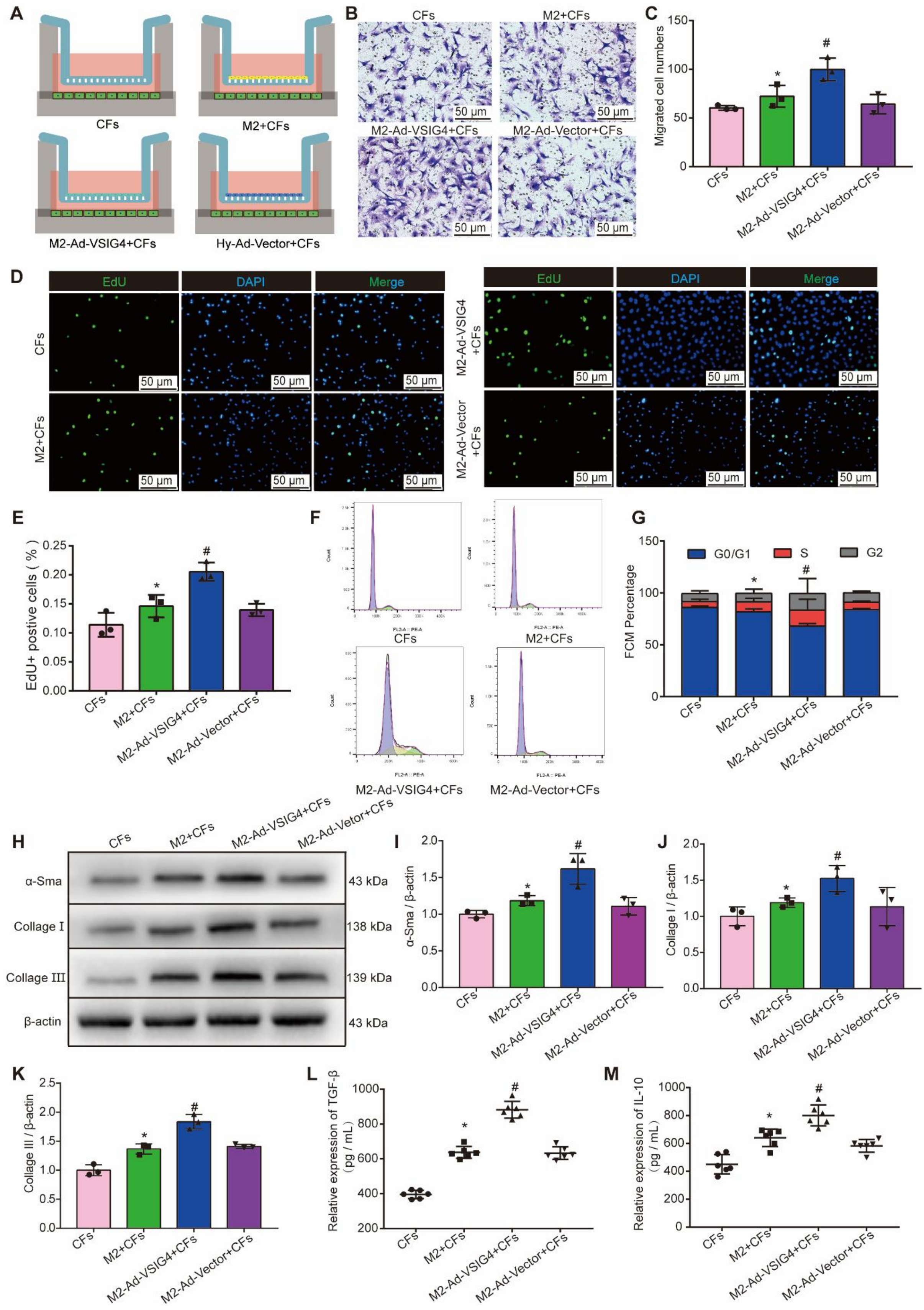

Supplementary Figure 6. Role of VSIG4 in the regulation of the proliferation

and migration of cardiac fibroblasts (CFs) and their phenotypic conversion to myofibroblasts. A, Co-culture model of M2 macrophages overexpressing VSIG4 cultured with CFs. **B,** Representative images of crystal violet staining for cell migration. **C,** Quantitative analysis of migrating cells (n = 3, $^{*}P < 0.05$ vs. CFs group, $^{\#}P < 0.05$, vs. M2+ CFs group; Scale bar, 50 μm). **D,** Representative EdU fluorescence images. **E,** Quantitative analysis of the proportion of EdU-positive cells (n = 3, $^{*}P < 0.05$ vs. CFs group, $^{\#}P < 0.05$ vs. M2+ CFs group; Scale bar, 50 μm). **F,** Representative images of cell cycle analysis. **G,** Quantitative analysis of cells in the G0/G1, S, and G2 phases (n = 3, $^{*}P < 0.05$ vs. CFs group, $^{\#}P < 0.05$ vs. M2+ CFs group). **H-K,** Representative western blot results of three independent experiments and quantification of αSMA, collagen I, and collagen III expression in CFs co-cultured with M2 overexpressing VSIG4 (n=3, $^{*}P < 0.05$ vs. CFs group, $^{\#}P < 0.05$ vs. M2+ CFs group). **L,** Quantification of TGF-β expression in the cell co-culture medium. **M,** Quantification of IL-10 expression in the cell co-culture medium (n = 6, $^{*}P < 0.05$ vs. CFs group, $^{\#}P < 0.05$ vs. M2+ CFs group).

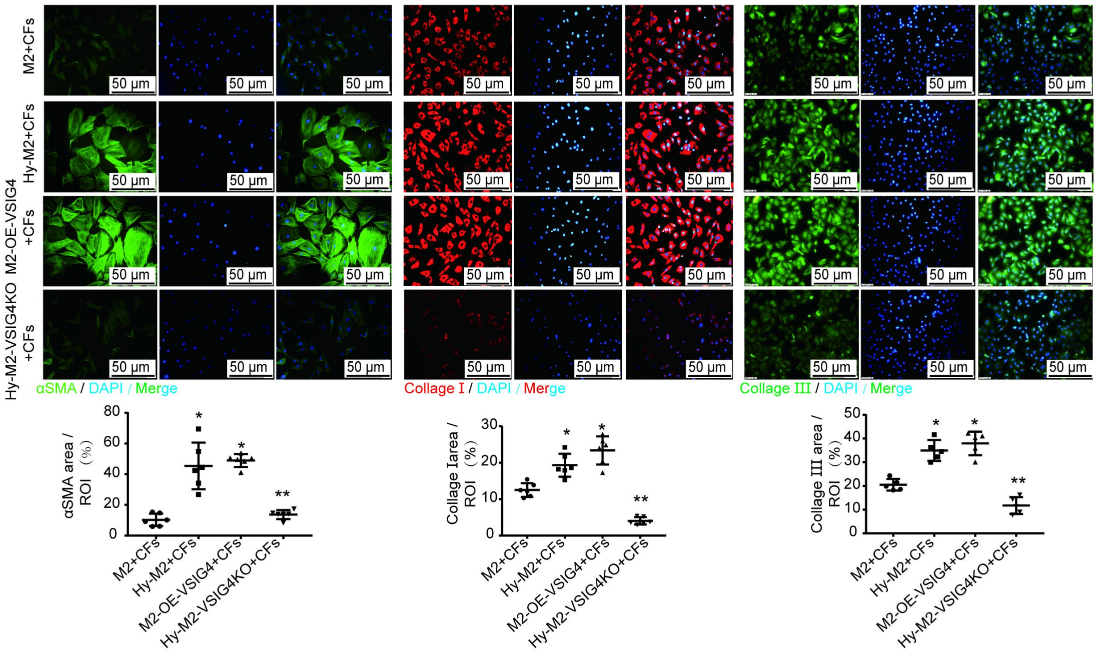

Supplementary Figure 7. Hypoxia mediates the effects of M2 macrophages on the expression of α-SMA, collagen I, and collagen III in cardiac fibroblasts (CFs) via VSIG4. Upper panel: Representative immunofluorescence staining of α-SMA (green), collagen I (red), and collagen III (green) in CFs (Scale bar, 50 μm). Lower panel: Histogram showing the relative quantification of α-SMA, collagen I, and collagen III immunofluorescence (n = 6, $^{*}P < 0.05$, vs. the M2+CFs group; $^{**}P < 0.05$ vs. the Hy-M2+CFs group).

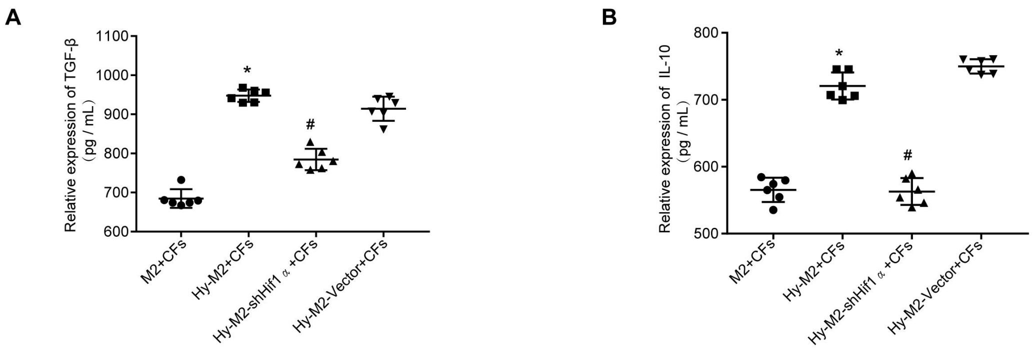

Supplementary Figure 8. Effects of Hif1α inhibition on the secretion of TGF-β and IL-10 in a co-culture system of hypoxic M2 (Hy-M2) and cardiac fibroblasts

(CFs). A, Quantification of TGF-β expression levels in the cell co-culture medium. **B,** Quantification of IL-10 expression in the cell co-culture medium (n = 6, $^{*}P < 0.05$ vs. M2+ CFs group, $^{\#}P < 0.05$ vs. Hy-M2+CFs group).
